# Modeling Synaptic Integration of Bursty and β Oscillatory Inputs in Ventromedial Motor Thalamic Neurons in Normal and Parkinsonian States

**DOI:** 10.1523/ENEURO.0237-23.2023

**Published:** 2023-12-08

**Authors:** Francesco Cavarretta, Dieter Jaeger

**Affiliations:** Department of Biology, Emory University, Atlanta, GA 30322

**Keywords:** basal ganglia, inhibition, motor cortex, potassium current, simulation, substantia nigra

## Abstract

The ventromedial motor thalamus (VM) is implicated in multiple motor functions and occupies a central position in the cortico-basal ganglia-thalamocortical loop. It integrates glutamatergic inputs from motor cortex (MC) and motor-related subcortical areas, and it is a major recipient of inhibition from basal ganglia. Previous *in vitro* experiments performed in mice showed that dopamine depletion enhances the excitability of thalamocortical (TC) neurons in VM due to reduced M-type potassium currents. To understand how these excitability changes impact synaptic integration *in vivo*, we constructed biophysically detailed mouse VM TC model neurons fit to normal and dopamine-depleted conditions, using the NEURON simulator. These models allowed us to assess the influence of excitability changes with dopamine depletion on the integration of synaptic inputs expected *in vivo*. We found that VM neuron models in the dopamine-depleted state showed increased firing rates with the same synaptic inputs. Synchronous bursting in inhibitory input from the substantia nigra pars reticulata (SNR), as observed in parkinsonian conditions, evoked a postinhibitory firing rate increase with a longer duration in dopamine-depleted than control conditions, due to different M-type potassium channel densities. With β oscillations in the inhibitory inputs from SNR and the excitatory inputs from cortex, we observed spike-phase locking in the activity of the models in normal and dopamine-depleted states, which relayed and amplified the oscillations of the inputs, suggesting that the increased β oscillations observed in VM of parkinsonian animals are predominantly a consequence of changes in the presynaptic activity rather than changes in intrinsic properties.

## Significance Statement

The ventromedial motor thalamus is implicated in multiple motor functions. Experiments *in vitro* showed this area undergoes homeostatic changes following dopamine depletion (parkinsonian state). Here, we studied the expected impact of these changes *in vivo*, using biophysically detailed modeling. We found that dopamine depletion increased firing rate in the ventromedial thalamocortical neurons and changed their responses to synchronous inhibitory inputs from substantia nigra pars reticulata. All thalamocortical neuron models relayed and amplified β oscillations from substantia nigra reticulata and cortical/subcortical inputs, suggesting that increased β oscillations observed in parkinsonian animals predominantly reflect changes in presynaptic activity.

## Introduction

Motor thalamus is a critical structure for multiple aspects of motor control (Bosch-Bouju et al., 2013). In rodents, it comprises the ventromedial (VM), the ventral-anterior (VA), and the ventrolateral nuclei (Kuramoto et al., 2011). In particular, VM and VA are the major recipients of inhibition from substantia nigra pars reticulata (SNR; Chevalier and Deniau, 1982; Kuramoto et al., 2011; Rovó et al., 2012), the output area of the basal ganglia. Excitatory inputs from primary and premotor cortices [motor cortex (MC)] play a key role in VM processing, supporting persistent motor preparatory activity in a closed loop (Z.V. Guo et al., 2017; K. Guo et al., 2018). *In vivo* experiments implicated VM in movement preparation and vigor control (Takahashi et al., 2021), while SNR mediates strong and temporally precise inhibition that controls movement direction and initiation in behaving animals (Morrissette et al., 2019; Catanese and Jaeger, 2021).

Parkinson’s disease (PD) is a neurodegenerative disorder that is primarily due to the death of dopamine neurons in the Substantia Nigra pars compacta. In rodents, dopamine depletion increases synchrony and bursting in the SNR (Lobb and Jaeger, 2015; Brazhnik et al., 2016; Willard et al., 2019). This maladaptive activity is conveyed to VM, and this pathway may be associated with deficits in movement selection and initiation (Kravitz et al., 2010; Morrissette et al., 2019; Takahashi et al., 2021). Moreover, local field potential (LFP) recordings showed increased β oscillations in SNR, MC, and VM of parkinsonian rats (Brazhnik et al., 2012, 2016; Nakamura et al., 2021), a typical hallmark of parkinsonian pathophysiology (Brown et al., 2001; Cassidy et al., 2002; Kühn et al., 2005; Sharott et al., 2018).

Previous experiments showed that dopamine depletion induced increased excitability in the VM of mice and increased their ability in generating rebound bursting on hyperpolarization (Bichler et al., 2021). Since there is little or no dopamine innervation to rodent VM (García-Cabezas et al., 2009), increased excitability is likely a homeostatic response due to increased inhibitory input from the SNR. As previously shown, such intrinsic excitability changes are broadly exhibited to compensate for a change in synaptic input balance (Desai et al., 1999; Turrigiano and Nelson, 2000). While experimental evidence suggests that the nigral synapses are well positioned to evoke rebound bursting in VM thalamocortical (TC) neurons (Bodor et al., 2008; Edgerton and Jaeger, 2014; Bichler et al., 2021), this hypothesis has not been tested *in vivo*. It remains unknown whether the effects of dopamine depletion on intrinsic VM properties may contribute to the generation of β oscillations in PD (Brazhnik et al., 2016), or exacerbate rebound bursting *in vivo*.

Because the contribution of intrinsic properties to firing patterns *in vivo* is hard to measure directly, we employed biophysically detailed modeling, using the NEURON simulator (Hines and Carnevale, 1997). Specifically, we fitted TC neuron models, replicating the different firing properties observed in normal and parkinsonian states (Bichler et al., 2021). Based on widely accepted thalamic literature (Reichova and Sherman, 2004; Bickford, 2016; Halassa and Acsády, 2016), we distinguished between driver (DRI), driver-like (DRI-l), and modulator (MOD) types of inputs. Specifically, we modeled four classes of afferent inputs to VM: glutamatergic MODs, approximating inputs from MC layer 6, glutamatergic DRI-l inputs from MC layer 5 and subcortical areas, and GABAergic inputs from SNR and reticular thalamic nucleus (RTN). Experimental evidence does not support the presence of excitatory drivers to VM (Rovó et al., 2012). Each afferent input replicated synaptic conductances and subcellular distributions observed experimentally (Bodor et al., 2008; Edgerton and Jaeger, 2014; Gornati et al., 2018), where the synapses were activated by artificial spike trains, replicating the activity during motor performance (Inagaki et al., 2022). This approach allowed us to control the firing patterns of the inputs, to reproduce either normal firing features or distinct firing features of the parkinsonian state. We then added varying amounts of synchronous bursting or β oscillations to these inputs. We found that TC neuron models in dopamine-depleted state responded at higher firing rate than TC neuron models in normal conditions to all *in vivo*-like input patterns. Synchronous nigral bursting was unable to evoke rebound bursting, as the synaptic excitation prevented sufficient hyperpolarization to de-inactivate T-type Ca^2+^ channels. However, synchronous nigral bursts still evoked a significant postinhibitory firing rate increase in TC neuron models, due to slow recovery of potassium currents on repolarization. This increase lasted longer in models fitted to dopamine-depleted conditions. Adding β oscillations in SNR inputs resulted in significant spike-phase locking in TC neuron model firing in both normal and parkinsonian states. This phase locking became stronger when excitatory inputs contained β oscillations at a 180-degree phase shift. These results suggest that the increased excitability induced by dopamine depletion does not affect β oscillations in VM TC neurons. In fact, in both normal and dopamine-depleted conditions our VM TC neuron models could amplify such oscillations.

## Materials and Methods

### Simulation

The morphology of a reconstructed TC neuron was divided in 40-μm-long compartments and used to construct multicompartmental biophysically detailed models with 11 Hodgkin–Huxley-style (HH) active conductances at varying densities (for details, see below, Neuron morphology and Intrinsic membrane properties).

Simulations were implemented in a fully integrated NEURON and Python environment (Hines and Carnevale, 1997). To fit the neuron models to physiological targets, we employed multiobjective optimization, based on genetic algorithms, using the BluePyOpt package (Van Geit et al., 2016). Here, each solution corresponds to a neuron model, encoded as an array of parameters associated with the intrinsic properties. The optimizer calculated model fitness by comparing the traces generated by each model against a set of features extracted from experimental recordings, simulating a battery of experiments with adaptive time steps, using the CVode solver for partial differential equations (for more details, see below, Fitting neuron models). The fitting process was executed on the supercomputer Expanse, managed by the San Diego Super Computer Center (SDSC), through the Neuroscience Gateway (NSG; http://www.nsgportal.org; Sivagnanam et al., 2013). Following model construction, simulations to evaluate model performance were executed using the NEURON version known as “CoreNEURON” (Kumbhar et al., 2019), and parallelized with the MPI4Py package. These simulations were executed on the supercomputer Expanse.

All simulation parameters such as temperature, reversal potentials of ion species, effects of ion channel blockers, and holding membrane voltage reflected the specifics of the experimental stimulation protocols and slice conditions (Table 1), while the number of active synapses reproduced experimentally observed values (Table 4). For each experiment, we calculated the values of reversal potential for sodium and potassium using the Nernst equation, accounting for the relative concentrations of ions in the artificial cerebrospinal fluid (aCSF) and pipette solutions. In some experiments, tetrodotoxin (TTX), a sodium channel blocker, was applied, and/or the pipette solution contained cesium, a potassium channel blocker. We accounted for their effects by turning off sodium and potassium HH-conductances in the simulations, respectively. To simulate *in vivo*-like conditions, we adjusted the temperature to 37°C (Figs. 3-8), which is higher than any simulated *in vitro* experiments (cf. 24–34°C; see Table 1). Increasing the temperature speeds up the kinetics of the ion channels in a way consistent with the Q10 rule (see below, Intrinsic membrane properties).

**Table 1:** Simulated experimental conditions

Reference	Temperature (°C)	V_hold_ (mV)	Experimental conditions
Bichler et al. (2021)	27	−69	E_Na_: 69 mV E_K_: −105 mV
Laudes et al. (2012)	24	−79.3	TTX application and pipette filled with cesium blocking sodium and potassium channels, respectively
Gornati et al. (2018)	34	−68.4	TTX application blocking sodium channels E_K_: −107.1 mV
Edgerton and Jaeger (2014)	32	−64	E_Na_: 60.1 mV E_K_: −105.8 mV

In this work, the simulations were replications of real experiments, accounting for temperature, holding membrane voltage (V_hold_), and reversal potential of ion species (E_rev_), such as sodium (Na) and potassium (K). The holding membrane voltages were corrected for the junction potential.

### Code accessibility

The source code is publicly available on ModelDB and GitHub (https://github.com/FrancescoCavarretta/BGMT).

### Modeling process overview

**Figure 1.**
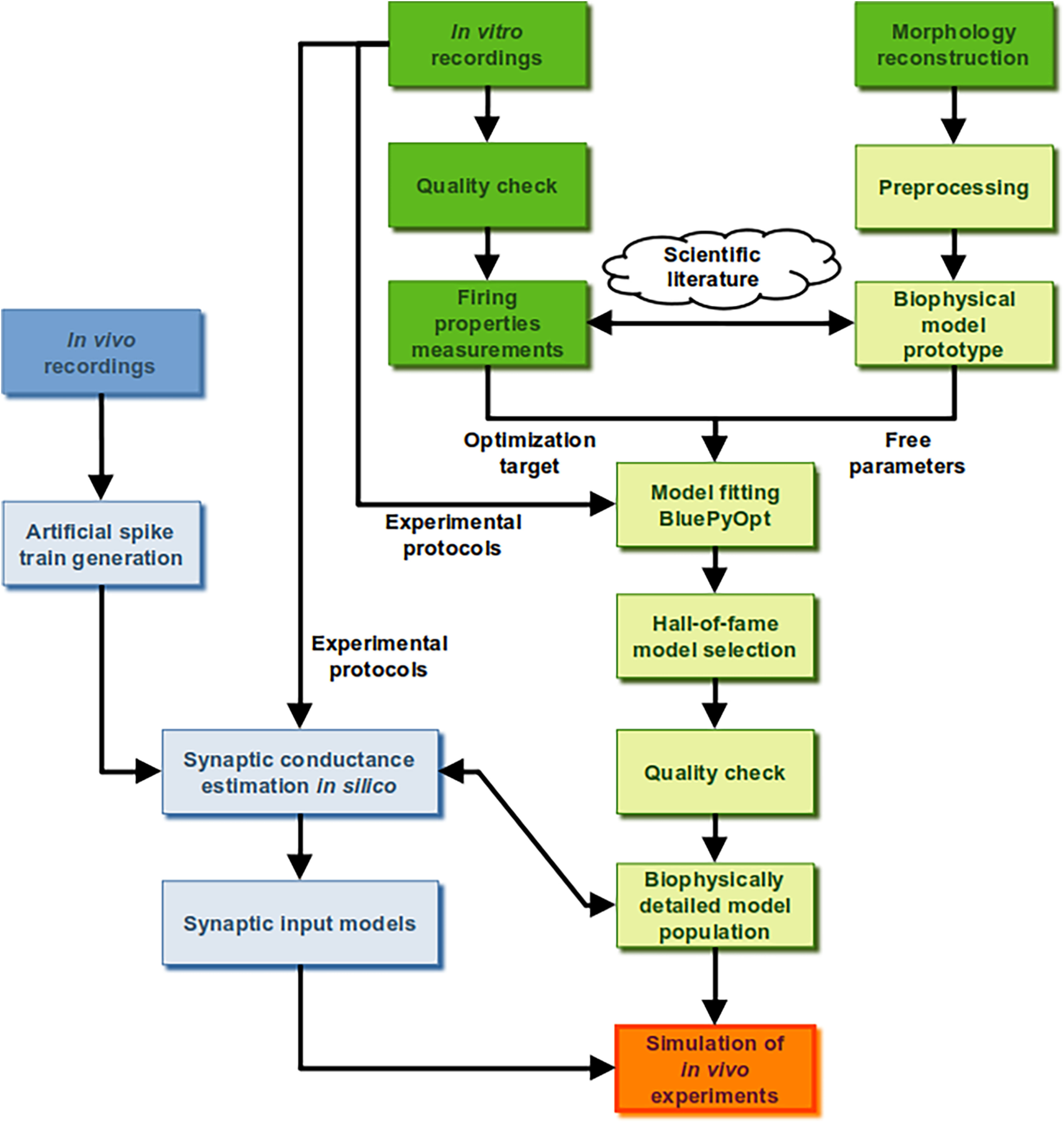
Simulation of *in vivo* firing activity of ventromedial motor thalamus in normal and parkinsonian states. The data-driven modeling pipeline for biophysically detailed simulations of *in vivo* conditions for thalamocortical neurons of ventromedial motor thalamus (VM) in normal and parkinsonian states. The pipeline comprises two branches, single cell (green) and synaptic afferent (blue) modeling, which converge on a single aim, the simulation of *in vivo*-like activity of mouse VM in normal and parkinsonian states (orange). Both branches start from the analysis of anatomical and electrophysiological data (dark green and dark blue), integrated with the information reported in literature (cloud).

We designed a data-driven modeling pipeline (Fig. 1) to build realistic TC neuron models from VM of mice in normal and parkinsonian states (green boxes) along with afferent inputs (blue boxes). We defined a biophysically detailed model for TC neurons, comprising a full three-dimensional morphology from the VM of mouse (see below, Neuron morphology) and 11 HH models of ion channels (see below, Intrinsic membrane properties), with subcellular distributions based on experimental data of thalamic and pyramidal cortical neurons (see below, Ion channel distributions). The model comprised multiple free parameters, with values determined by multiobjective optimization (see below, Template data taken from *in vitro* experiments and Fitting neuron models). We then modeled the main synaptic inputs to VM, reproducing the subcellular distributions, numbers of synaptic contacts, and unitary conductances observed experimentally (see below, Modeling synaptic inputs), with realistic levels of presynaptic activity (see below, Presynaptic activity: artificial spike train generation).

### Neuron morphology

We based our model on a full three-dimensional reconstruction of a TC neuron from the VM of mouse (id. AA0719; https://www.janelia.org/project-team/mouselight). For modeling purposes, we retained only the proximal 70-μm-long portion of the axon (i.e., axonal initial segment), approximated by two cylindrical compartments (diameter: 1.5 μm; length: 35 μm), as we did not further follow action potential propagation. The original reconstruction lacked estimates for dendritic diameters while the soma was represented by the cross-sectional perimeter only. We replaced the soma with a cylindrical section (diameter: 36.9 μm; length: 24 μm), yielding a cross-section surface of 293 μm^2^, consistent with the experimental estimates of VM TC neurons of rats (cf. 288 ± 13 and 298 ± 2 μm^2^; ±SE; Sawyer et al., 1989). To model the dendritic diameters, we defined a recursive formula based on Rall’s 2/3 Power Law:

If the dendrite has children:

$$diam={\left(Q_{1}+M_{1}^{}\overset{}{\underset{i\in \left\{1,2\right\}}{\sum }}diam_{child,i}^{1.5}\right)}^{1.5}$$

with M_1_ = 0.515, Q_1_ = 0.182.

Otherwise:

$$diam=Q_{2}-M_{2}\cdot depth$$

with M_2_ = 0.004, Q_2_ = 0.473, where *diam* indicates the dendritic diameter, *depth* indicates the number of branch points from soma, and *diam_child,i_* is the diameter of the i^th^ child. Additionally, for primary dendrites only, the diameter tapers with the distance from soma (*d*):

$$diam\left(d\right)=diam\cdot max\left\{DIAM_{min},C_{3}+A_{3}\cdot exp\left(-B_{3}d\right)\right\}$$

with A_3_ = 0.861, B_3_ = 0.045, C_3_ = 0.858, DIAM_min_ = 1, with *diam* defined as above. Figure 3*B* shows the resulting dendritic diameters.

The values of the free parameters (i.e., M_*_, Q_*_, A_3_, B_3_, C_3_, and *DIAM_min_*) were estimated by calculating the least square regression curves from partial histologic reconstructions of VM TC neurons (*n *=* *5) of mouse obtained in our laboratory, using biocytin and Neurolucida. Specifically, we estimated *Q_1_* and *M_1_* by linear regression between the diameters of parent dendrites versus the sum of the diameters of children to the 1.5 power; *Q_2_* and *M_2_* by linear regression of diameters versus branch depth for the dendrites that did not have branches; *C_3_*, *B_3_*, and *A_3_* were estimated by fitting the exponential function *C_3_ + A_3_ exp(-B_3_d)* on the diameters of the primary dendrites normalized to the average as a function of the distance from soma.

### Intrinsic membrane properties

NEURON models require the specification of passive properties, such as specific membrane specific membrane conductivity (g; i.e., inverse of resistivity), axial resistivity (r_i_; i.e., intracellular or cytoplasmic resistivity), specific membrane capacitance (c_m_), and resting potential (V*_Rest_*). In our models, their values were uniform across all sections. In particular, c_m_ was a constant using the canonical value (1 μF/cm^2^; Gentet et al., 2000), while the others were treated as free parameters, estimated by our genetic algorithm (see below, Fitting neuron models).

**Figure 2.**
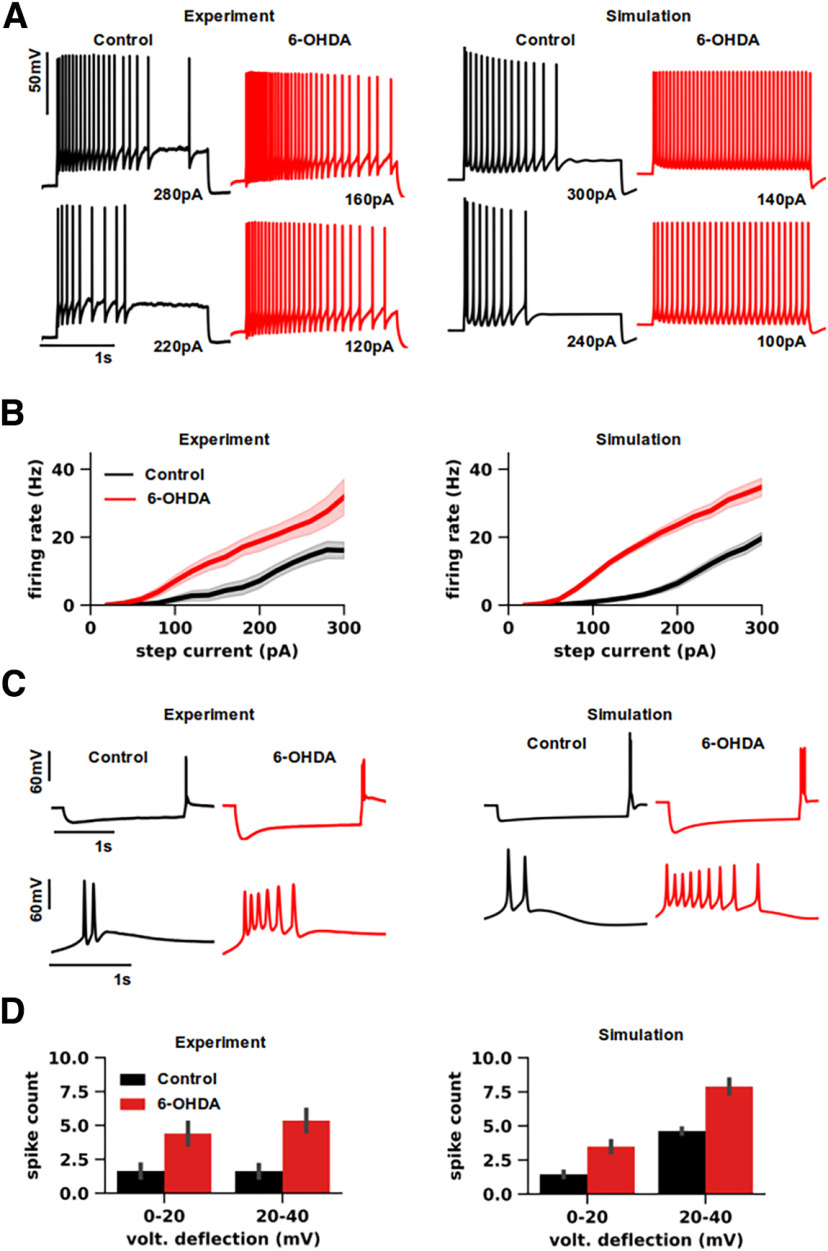
Experimental and simulated responses of thalamocortical neurons from ventromedial motor thalamus in normal and parkinsonian states. ***A***, Representative neuron responses to increasing depolarizing current injections. The baseline membrane potential was depolarized to −69 mV by a bias current (Control: 93 pA and 6-OHDA: 67 pA). The bias current inactivated T-type Ca^2+^ channels and thus enabled tonic firing generation. Left, Whole-cell recordings from ventromedial thalamus (VM) of control mouse (black) and 20 weeks after 6-OHDA injection (red). Right, Simulated neuron responses of thalamocortical (TC) neurons from VM of mouse in normal (black) and parkinsonian (red) models. Additional experimental and simulation traces are shown in Extended Data Figure 2-1. Both experimental and simulated recordings display variability in different action potential (AP) properties, such as AP amplitude, after-hyperpolarization (AHP) depth, and firing rate adaptation. The AP properties of experimental and simulation traces are statistically indistinguishable in both normal and parkinsonian conditions (Mann–Whitney, *p* > 0.04), except the AP amplitudes, as shown in Extended Data Figure 2-2. ***B***, The f-I curves for TC neurons of VM in normal (black) and parkinsonian (red) states (±SE). Dopamine depletion increased the firing rate and shifted f-I curves to the left (Wilcoxon, *p* < 0.001) in both experiments (Control: *n* = 9 and 6-OHDA: *n* = 7 cells; left) and simulations (Control: *n* = 16 and 6-OHDA: *n* = 17 models; right). The step current (20–300 pA with 20-pA increments) was delivered on top of the bias current (same as in ***A***). ***C***, Representative neuron responses to hyperpolarizing current injection (−200 pA) on top of a bias current (same as in ***A***), evoking rebound bursting at the offset of the step current. Left, Whole-cell recordings from VM of control mouse (black) and after 6-OHDA treatment (red). Right, Simulation of the same stimulation protocol and experimental conditions with normal (black) and parkinsonian (red) neuron models. ***D***, Bar graph pairs representing the average rebound spike count (±SE) sorted by the amplitude of voltage deflection reached during hyperpolarizing current steps (−50 to –200 pA with 50-pA increments) in controls (black) and in parkinsonian (red) conditions. Rebound spike count was significantly higher for parkinsonian neurons than control in both experiments (Mann–Whitney, 20–40 mV: *p* = 0.0125; 40–60 mV: *p* = 0.005; Control: *n* = 9 and 6-OHDA: *n* = 7 cells; left) and simulations (Mann–Whitney, 20–40 mV: *p* < 0.001; 40–60 mV: *p* < 0.001; Control: *n* = 16 and 6-OHDA: *n* = 17 models; right).

The active membrane properties of our models consisted of 11 HH-conductances (Extended Data Table 3-1), with states dependent on the membrane potential and/or intracellular Ca^2+^ concentration. They were transient (Na_T_) and persistent (Na_P_) sodium currents; delayed rectifier (K_DR_), A-type (K_A_), delaying (K_D_), and M-type (K_M_) potassium currents; H-type nonspecific cation current (I_H_); T-type (Ca_T_) and L-type (Ca_L_) Ca^2+^ currents; small conductance (SK) and big conductance (BK) Ca^2+^-dependent potassium currents. Most HH-conductances were specified as used in a previous model of ventrobasal TC neuron (Na_T_, K_DR_, K_A_, I_H_, Ca_T_, Ca_L_, SK; Iavarone et al., 2019), while K_M_ was specified as used in a previous model of VM TC neuron (Bichler et al., 2021). We derived the Na_P_ dynamics from the Na_T_ model (Iavarone et al., 2019), decreasing the half-values of activation and inactivation curves by 14 mV, consistent with experimental estimates from pyramidal cells (Hu et al., 2009). We based the K_D_ model on the dynamics of the shaker-related potassium channels observed in TC neurons of rats (Lioudyno et al., 2013), where the effects of these channels delayed the action potential (AP) onset (Lioudyno et al., 2013), similarly to the effects of K_D_ currents observed in pyramidal neurons (Storm, 1988). We added the BK to induce firing rate adaptation in TC neurons (Ehling et al., 2013), observed also in VM (Fig. 2; Bichler et al., 2021). The BK dynamics were voltage and Ca^2+^ -dependent (Rothberg and Magleby, 2000), and accounted for the dependence on temperature (Q_10_ = 2.5; Yang and Zheng, 2014).

10.1523/ENEURO.0237-23.2023.f2-1 Extended Data Figure 2-1 Diversity of experimental and simulated responses of thalamocortical neurons from ventromedial motor thalamus in normal and parkinsonian states. Neuron responses to increasing depolarizing current injections for different neurons (*n* =* *3) and models (*n* =* *3), under the same experimental conditions as in Figure 2*A*. Both experimental and simulated recordings display variability in different action potential (AP) properties, such as AP amplitude, AHP depth, AP accommodation. Download Figure 2-1, TIF file.

10.1523/ENEURO.0237-23.2023.f2-2 Extended Data Figure 2-2 Comparison of action potential properties between experimental and simulation traces. Experimental and simulation traces displayed similar firing properties. A subset of experimental and simulation traces is shown in Figure 1*A* and Extended Data Figure 1-1. Each data point (black) is associated with a single TC neuron (Control: *n* = 9 and 6-OHDA: *n* = 7 neurons) and TC neuron model (Control: *n* = 16 and 6-OHDA: *n* = 17 models). We compared after-hyperpolarization (AHP) depth (top, left), action potential (AP) amplitude (top, right), firing (rate) adaptation (bottom, left), and sag amplitudes (bottom, right) of TC neurons (Control: *n* = 9 and 6-OHDA: *n* = 7 neurons; blue) and TC neuron models (Control: *n* = 16 and 6-OHDA: *n* = 17 models; orange). The same firing properties were considered as a target during the model fitting (see also Materials and Methods) and are described in Table 2. We used protocol 1 (i.e., the same stimulation protocol as in Figure 1*A*; for details, see Materials and Methods) to measure AHP depth, AP amplitude, and firing adaptation, while we used protocol 3 for sag amplitudes, in both experiments and simulations. The values of AHP depth and AP amplitude were estimated using threshold amplitudes of step current, while we used the minimal current evoking at least five APs for firing rate adaptation. As the threshold current depends on the input resistance of neurons, it was estimated for each real neuron and model. To measure the sag amplitudes, we used a hyperpolarizing step current (−200 pA). Comparing experimental and simulation traces, we found that most firing features were statistically indistinguishable in both normal and parkinsonian conditions (Mann–Whitney, *p* > 0.04), except the AP amplitudes (Mann–Whitney, Control: *p* < 0.01; 6-OHDA: *p* < 0.001), which were lower in simulations than experiments (Control: 21%; 6-OHDA: 33%). Download Figure 2-2, TIF file

**Table 2:** Summary of experimental protocols and targets for neuron model evaluation during the fitting process

**List of optimized performance measures**
**Protocol 1:** **For control conditions measures were taken with: 2-s-long Current injection steps of 2 s: 200 pA, 240 pA, 280 pA, Bias: 93 pA** **For Parkinsonian conditions measures were taken with: 2-s-long Current injection steps of 2 s: 120 pA, 180 pA, 240 pA, Bias: 67 pA**
Avg. action potential (AP) amplitudes [AP_amplitude]
Avg. slow after-hyperpolarization (AHP) depth [AHP_depth]
Avg. AP width [AP_width]
AP_count
First-spike latency
Instantaneous firing rate calculated as the inverse of the first ISI (1/ms) [inv_first_ISI]
Instantaneous firing rate calculated as the inverse of the second ISI [inv_second_ISI]
Instantaneous firing rate calculated as the inverse of the last ISI [inv_last_ISI]
Average difference between consecutive ISIs normalized to their sum [adaptation_index2]
Voltage baseline before current step [voltage_base]
Voltage baseline after current step [voltage_after_stim]
Spontaneous AP count before current step [AP_count_before_stim]
Spontaneous AP count after current step [AP_count_after_stim]
Difference between maximum and minimum AP amplitudes [max_amp_difference]
Ratio between AP counts evoked within the first and second half of the current step [clustering_index]
Avg. fast AHP depth [fast_AHP]
**Protocol 3:** **All Conditions: 2-s-long Current injection Step: -200 pA**
1st AP amplitude [AP1_amp_rev]
2nd AP amplitude [AP2_amp_rev]
time_to_first_spike
inv_first_ISI
inv_second_ISI
inv_last_ISI
voltage_base
voltage_after_stim
AP_count_before_stim
AP_count_after_stim
Relative difference (in percentage) between voltage steady state and voltage deflection, both measured as the difference from baseline [sag_amplitude]
Difference between minimum voltage value and baseline [voltage_deflection]
**Protocol 4:** **All Conditions: 0.5-ms-long Single Pulse of –1 nA**
Membrane time constant [decay_time_constant_after_stim2]
**Protocol 5:** **All Conditions: 100-ms-long Current Step of –10 pA**
Ratio between voltage deflection and current amplitude [input_resistance]

Labels in square brackets indicate the parameter identifier used in the source code.

Consistent with previous experimental studies (Womack et al., 2004), intracellular Ca^2+^ was structured in three separate microdomains, associated with distinct decay time constants. In particular, the Ca^2+^ flowing through Ca_L_ channels interacts with two microdomains that activate SK and BK channels separately, while Ca^2+^ flowing through Ca_T_ channels interacts with a third microdomain that does not activate other ion channels. In our models, the Ca^2+^ concentration of each microdomain determined the reversal potential for the related Ca^2+^ channel, calculated with the Goldman–Hodgkin–Katz flux equation.

### Ion channel distributions

Based on experimental data of TC neurons (Budde et al., 1998; Williams and Stuart, 2000), we modeled the subcellular distributions of ion channels as follows:

- The conductance density of voltage-dependent potassium channels (i.e., K_DR_, K_A_, K_M_, K_D_) is uniform throughout the somatodendritic shaft.
- The conductance density of sodium channels (i.e., Na_T_, Na_P_) decreases at each branch point.
- For T-type Ca^2+^ channels (i.e., Ca_T_), the conductance density is double as that of the soma for primary dendrites, and half of the soma for all the other dendrites.
- The conductance density of L-type Ca^2+^ channels (i.e., Ca_L_) decreases to half of the soma within the proximal 10-μm-long portion of primary dendrites, and to a third in all other dendritic locations.

As the subcellular distribution of small-conductance and big-conductance Ca^2+^-dependent potassium channels (i.e., Ca_L_, SK, BK) is unknown in TC neurons, and given the lack of data for the axon initial segment (AIS) of TC neurons, we modeled their distributions on data from pyramidal cells (Bowden et al., 2001; Hu et al., 2009; Battefeld et al., 2014):

- As Ca_L_ and the Ca^2+^-dependent conductance densities (i.e., SK) are co-localized in pyramidal cells, we used the same subcellular distributions for SK and BK, while these channels were absent in the axon.
- Na_T_ and Na_P_ conductance densities can be up to 19-fold higher than in the soma in the proximal and distal halves of the AIS, respectively.
- K_M_ conductance density in the distal half of the AIS can be up to 50 times higher than in the soma.

In the absence of specific information, we made the following assumptions:

- H-type conductance density is uniform along the somatodendritic arbor but absent in the AIS.
- K_DR_ conductance density can be up to 5-fold higher in the proximal half of the AIS than in the soma to compensate for the effects of the high Na_T_ conductance density, thus enabling full action potential repolarization.

### Template data taken from *in vitro* experiments

We selected a subset of whole-cell recordings obtained from a previous study *in vitro* (Bichler et al., 2021), performed on adult mice of either sex in normal conditions and after unilateral 6-OHDA injections placed in the median forebrain bundle (Control: *n* = 9; 6-OHDA: *n* = 7). These recordings were obtained with different experimental protocols:

- Protocol 1: 2-s-long depolarizing current steps (20–300 pA with 20-pA increments) on top of a bias current that held the membrane potential at −69 mV (Control: 93 pA; 6-OHDA: 67 pA), to estimate f-I curves.
- Protocol 2: 2-s-long hyperpolarizing current steps (from –200 to –50 pA with 50-pA increments) on top of a bias current (same as in protocol 1), to estimate the rebound spike count versus current relationship.
- Protocol 3: 2-s-long hyperpolarizing steps (–200 to –50 pA with 50-pA increments; without bias current), to measure sag amplitude and evoke rebound bursting versus current relationships.
- Protocol 4: single pulse of –1 nA and 0.5 ms of duration, to measure membrane time constant.
- Protocol 5: 100-ms-long current step of –10 pA, to estimate input resistance.

To measure firing properties from simulation and experimental traces, we used a custom version of the eFEL package (https://github.com/BlueBrain/eFEL). In particular, the targets for model performance were constituted by membrane and firing properties extracted from our experimental recordings obtained with protocols (see Table 2 for a complete list).

### Fitting neuron models

We define a TC neuron model comprising 33 free parameters, including the conductance densities along with the decay time constants of intracellular Ca^2+^ concentration (see above, Intrinsic membrane properties). We also added voltage shift variables and multiplicative factors as free parameters to the equations describing the dynamics of Na_T_, K_DR_, K_M_, and BK currents, with different effects on the firing properties generated by our models. Specifically, we added:

- Multiplicative factors to the equations of the rates for activation and inactivation of Na_T_ and K_DR_, fixing the ranges in a way that slowed down their dynamics, and thus regulated the AP width.
- A multiplicative factor to the equation of the rates for the activation of K_M_, fixing the ranges in a way that slows down the de-activation, and thus calibrated the contribution to firing rate adaptation.
- A voltage shift variable to the equations of activation and inactivation, along with their rates, for K_D_, fixing the range in a way that increased the impact of the channel on the first-spike latency, yielding half-values of activation and inactivation more similar to the values observed in pyramidal cells (Storm, 1988).
- Multiplicative factors and shift variables to the equations describing activation and inactivation, along with their time constants, for BK, mimicking the effects of β subunits expression on the channel dynamics (Behrens et al., 2000; Contreras et al., 2012), to modulate the impact of this channel on firing rate adaptation.

The values of the free parameters were determined by multiobjective optimization, using the BluePyOpt toolkit (Van Geit et al., 2016) to fit model traces to a set of target features (Table 2). These features described membrane potential dynamics (e.g., action potential amplitudes, after-hyperpolarization depth, spike count, firing adaptation index, and so on) in response to current injection paradigms and were extracted from our published whole-cell recordings in normal and parkinsonian states (Bichler et al., 2021).

Model candidates in normal and parkinsonian conditions were obtained with 45 and 15 optimization sessions, respectively, using a different random seed for each session, with 100 individuals and 100 generations per session. Technically, the optimizer minimized the error associated with each model as measured by the difference between electrophysiological features from simulations and experimental traces. The overall fitness of a given model resulted from the sum of the absolute errors associated with feature differences (passive features and active features; Table 2), each calculated as the deviation from the experimental mean normalized to the experimental standard deviation. To measure the firing properties of each model, the optimizer ran a battery of simulations reproducing experimental traces (Table 2). For final model selection, we first made a hall-of-fame as the population of models for which all errors fell within three standard deviations from mean (Control: *n* = 686; 6-OHDA: *n* = 4900). Of these models, we retained the ones that passed additional quality checks. First, we compared the entire f-I curves, obtained with protocol 1, along with the rebound spike count versus current relationships, obtained with protocols 2 and 3, retaining the hall-of-fames that yielded spike counts below three standard deviations from the experimental mean throughout the entire range of stimulation (Control: *n* = 78; 6-OHDA: *n* = 922). Second, we selected the models that replicated the effects of XE991 application (10–20 μm; Bichler et al., 2021), simulated by decreasing the M-type conductance density by 70% (Yeung and Greenwood, 2005). In the experiments, XE991 application in normal condition decreased the rheobase by ∼80 pA and shifted the f-I curves to the left, while the application did not alter the response in parkinsonian condition. For each model, we then compared the f-I curves with and without XE991, retaining normal and parkinsonian models generating distinguishable (Wilcoxon, *p* < 0.1; with shift in rheobase < 100 pA) and indistinguishable (Wilcoxon, *p* ≥ 0.9; with no shift in rheobase) curves (Control: *n* = 39; 6-OHDA: *n* = 21). Third, by inspecting traces, we rejected models that generated a nonphysiological after-hyperpolarization, with peaks below baseline (Control: *n* = 28; 6-OHDA: *n* = 19). Fourth, we tested the models with realistic synaptic inputs (see below, Modeling synaptic inputs), retaining the models that generated firing rates between 5 and 90 Hz (Control: *n* = 16; 6-OHDA: *n* = 17). The distributions of the parameters for normal and parkinsonian models are described in Table 3.

**Table 3:** Distributions of parameters for thalamocortical neuron models in normal and parkinsonian states

	Normal	Parkinsonian
Passive properties		
r_i_ (Ω·cm)	156.35 ± 8.45	165.27 ± 9.1
V_rest_ (mV)	−77.6 ± 1.31	−73.3 ± 1.76
g (S/cm^2^)	13.29 ± 2.76	6.64 ± 1.91
Active properties		
Na_T_		
T_m_	0.55 ± 0.07	0.66 ± 0.05
T_h_	0.48 ± 0.06	0.36 ± 0.06
g (S/cm^2^)	6235.45 ± 1234.67	9995.27 ± 1229.78
g_extra_	19,3703 ± 22,051	184,569 ± 26,511.8
Na_P_		
g (S/cm^2^)	685 ± 25.11	650.59 ± 32.54
g_extra_ (S/cm^2^)	14,818.8 ± 520.36	12,377.9 ± 1130.27
K_DR_		
T_n_	0.72 ± 0.05	0.64 ± 0.03
g (S/cm^2^)	14,715.1 ± 1866.8	16,559.7 ± 1589.31
g_extra_ (S/cm^2^)	83,856.7 ± 11,398.4	57,216.8 ± 10,598.8
K_A_		
g (S/cm^2^)	739.57 ± 126.93	1153.67 ± 126.86
K_D_		
V_1/2, shift_ (mV)	14.16 ± 2.58	23.43 ± 2.48
g (S/cm^2^)	129.15 ± 18.91	142.91 ± 22.69
K_M_		
T_m_ (*)	8.86 ± 0.28	4.83 ± 0.57
g (S/cm^2^; *)	306.76 ± 29.24	9.69 ± 2.46
g_min_ (S/cm^2^)	12.97 ± 1.56	18.51 ± 1.05
g_extra_ (S/cm^2^)	16,629.2 ± 1982.26	18,731.6 ± 1769.36
I_H_		
g (S/cm^2^)	40.97 ± 5.1	55.57 ± 5.78
Ca_T_		
g (S/cm^2^; *)	38.14 ± 3.08	82.54 ± 5.02
Ca_L_		
p_1_ (cm/S)	93.78 ± 30.2	119.8 ± 44.8
p_2_ (cm/S)	498.43 ± 80.7	534.23 ± 86.29
SK		
g (S/cm^2^)	433.52 ± 122.8	736.62 ± 188.64
BK		
V_1/2, shift_ (mV)	−62.22 ± 7.53	−47.81 ± 7.75
T_m_	6.23 ± 1.04	5.58 ± 0.75
g (S/cm^2^)	10,450.4 ± 1364.94	8339.23 ± 1397.8
Ca^2+^ buffer		
L_1_		
τ (ms)	31 ± 5.21	23.79 ± 4.56
δ (1/μm)	2.2 ± 0.55	1.62 ± 0.51
L_2_		
τ (ms)	1639.33 ± 211.96	1682.75 ± 189.93
δ (1/μm)	9.75 ± 1.42	10.42 ± 1.18
T		
τ (ms)	52.7 ± 6.12	48.06 ± 6.66
δ (1/μm)	17.19 ± 2.56	16.54 ± 1.63

The confidence interval is described in ±SE. Passive properties: r_i_: specific intracellular resistivity; V_rest_: resting membrane potential; g: specific membrane conductivity. Active properties: T_m_, T_h_, T_n_: multiplicative factors for activation (*m*, *n*) and inactivation (*h*) time constants of ion channel; g: specific conductance density of ion channel; g_extra_: additional conductance in the axon initial segment for a ion channel; g_min_: minimal conductance density; V_1/2, shift_: shift of activation and/or inactivation curves for a ion channel; p_1_, p_2_: permittivity of ion channel; Ca^2+^ buffers: τ: decay time constant of intracellular Ca^2+^; δ: inverse shell depth. Parameters with distributions that are statistically different (Mann–Whitney, *p* < 0.01) between normal and parkinsonian models are marked with asterisks (*). The equations describing the active membrane properties are shown in Extended Data Table 3-1.

### Modeling synaptic inputs

We modeled four groups of synaptic inputs: glutamatergic driver-like (DRI-l) inputs, glutamatergic modulators (MOD), GABAergic inputs from substantia nigra reticulata (SNR), and GABAergic inputs from reticular thalamic nuclei (RTN), each defined by postsynaptic location, numbers of terminals, and unitary conductance (Table 4), representing the presynaptic activity by artificial spike trains with realistic firing rates and irregularity (i.e., coefficient of variation of interspike interval).

**Table 4:** Estimates of unitary synaptic conductance for each synaptic input to ventromedial motor thalamus

	Simulation	Experiment	Reference
MOD	g_syn_: 2.2 nS mEPSC: −28.6 ± 0.2 pA	mEPSC: −28.4 ± 2.1 pA	1
RTN	g_syn_: 0.7 nS mIPSC: 26.6 ± 0.6 pA	mIPSC: 24.43 ± 0.75 pA	1
SNR	g_syn_: 4.8 nS mIPSC: 47.3 ± 21.5 pA with 1 active terminal	IPSC: 47.2 pA, i.e., 4.25 times the minimum IPSC (11.1 pA)	2
DRI-l	g_syn_: 1.3 nS EPSC: 126.7 ± 10.3 pA with 4 active terminals	EPSC: 123.7 pA, i.e., 75% of −165.0 ± 40.2 pA, with ∼4 active terminals	3

Summary of estimated unitary conductance (g_syn_) for each group of synaptic inputs to ventromedial motor thalamus. MOD, modulators; RTN, reticular thalamic nuclei; SNR, substantia nigra reticulata; DRI-l, driver-like inputs. References: 1, Laudes et al. (2012); 2, Edgerton and Jaeger (2014); 3, Gornati et al. (2018). Conductance values yielded amplitudes of IPSC and EPSC (±SE) that match the amplitude measured in patch-clamp experiments on ventrobasal (Laudes et al., 2012) and ventromedial (Edgerton and Jaeger, 2014; Gornati et al., 2018) thalamic nuclei. In one experiment (Laudes et al., 2012), one reported the amplitudes for miniature IPSCs (mIPSCs) and EPSCs (mEPSCs).

Anatomical studies showed that vGluT1+ and vGluT2+ terminals in VM, which originate from layer 5 of cortex and subcortical regions, respectively, have small to medium size terminals, while large size terminals were not observed (Rovó et al., 2012), suggesting that the big excitatory driver synapses observed in other thalamic nuclei may be absent in VM. On the other hand, cerebellar terminals display “driver-like” physiology, whose activation evoked smaller EPSCs than classic drivers (Gornati et al., 2018). Therefore, we stayed with identical properties for all driver-like inputs to VM, with parameters matching cerebellothalamic terminals in VM.

Specifically, excitatory synapses comprised AMPA and NMDA components (Cavarretta et al., 2018), with a reversal potential of 0 mV, and NMDA/AMPA ratios of 0.6 for drivers and 1.91 for modulators as estimated from ventrobasal thalamus (Miyata and Imoto, 2006). For inhibitory synapses, we set the decay time constant to 14 ms (at 32°C; Edgerton and Jaeger, 2014), reversal potential to –81 mV (Ulrich and Huguenard, 1997), and Q_10_ to 2.1 (Otis and Mody, 1992). For each group of synapses, we determined subcellular distributions and numbers of contacts. We fixed the proportion of DRI-l in relation to MOD terminals to 10%, consistent with previous anatomic studies on drivers and modulators (Van Horn et al., 2000; Van Horn and Sherman, 2007). The DRI-l terminals were located at a distance from the soma that reproduced the distribution observed for deep cerebellar nuclei terminals in VM (Gornati et al., 2018). For the other synaptic inputs, we estimated the densities of synaptic terminals along the circumference of single dendritic sections obtained by electron microscopy (EM) of mouse VM (*n* =* *2; Y. Smith, unpublished data). Specifically, we analyzed EM pictures of transversal dendrite profiles, obtained with magnifications between 20,000 and 40,000. The dendrites were then divided in three brackets based on cross-section diameters, namely small (0–0.5 μm; *n* =* *222), medium (0.5–1 μm; *n* =* *220), and large (>1 μm; *n* =* *25). According to previous studies (Swain et al., 2020), synaptic terminals were categorized as asymmetric (AS) terminals, originating from MC, type-1 symmetric (S1) terminals, originating from RTN, and type-2 symmetric (S2) terminals, originating from SNR. Only terminals that showed an active zone at the level of a given EM section were counted. From these counts, we calculated the density of each type of terminal as density of terminals around the circumference of the dendritic profiles. We then assumed that independent samples of terminals along the dendrite would be obtained at distances equal to the average diameter of terminals, which is ∼0.8 μm for MOD and RTN terminals (Y. Smith, unpublished data), and 2.8 μm for SNR terminals (Bodor et al., 2008). Thus, the cross-sectional density of synapses around the circumference of dendrites was applied for every 0.8 μm (MOD and RTN) or 2.8 μm (SNR) of dendritic length in the model. Finally, we added SNR terminals contacting the TC neuron soma as a proportion of the dendritic ones (Bodor et al., 2008).

After defining the subcellular distributions, we estimated the unitary synaptic conductance for each group of synapses. To this end, we designed an optimizer that determined the optimal values matching the postsynaptic current amplitudes observed experimentally (Table 4, experiment). The optimizer explored the range of conductance values by using a convergence algorithm, testing each value by running simulations that reproduced the voltage-clamp experiments (Table 4, simulation).

We did not model short-term plasticity (STP) for any synapses, as an experimental characterization of STP parameters in VM is lacking. Moreover, a previous modeling work suggested that the main effect of short-term depression in a simulation with steady state rates of input is equivalent to a downscaled unitary conductance (Abbasi et al., 2017). We thus reduced the unitary conductances to approximate the effects of short-term depression in the DRI-l inputs (Gornati et al., 2018). Specifically, we considered an EPSC amplitude reduced by 25% as a target for estimating the conductance peak of the DRI-l synapses (see Table 4, experiment) so accounting for the paired-pulse ratio observed experimentally (Gornati et al., 2018).

By applying the subcellular distributions of synaptic terminals described above to the morphology (AA0719; MouseLight Archive), we obtained estimates of the number of terminals for each group of synapses (MOD: 3625; DRI-l: 350; SNR: 25; RTN: 400). In our simulations, we decreased the MOD terminals to 1450, i.e., ∼40% of the estimated value, accounting for the proportion of silent and nonsilent pyramidal neurons observed in layer 6 of MC of cats (Sirota et al., 2005). This configuration yielded a basal firing rate of 18.6 ± 9.4 Hz (±SD) in normal conditions, consistent with the values observed experimentally (Inagaki et al., 2022).

### Presynaptic activity: artificial spike train generation

**Figure 3.**
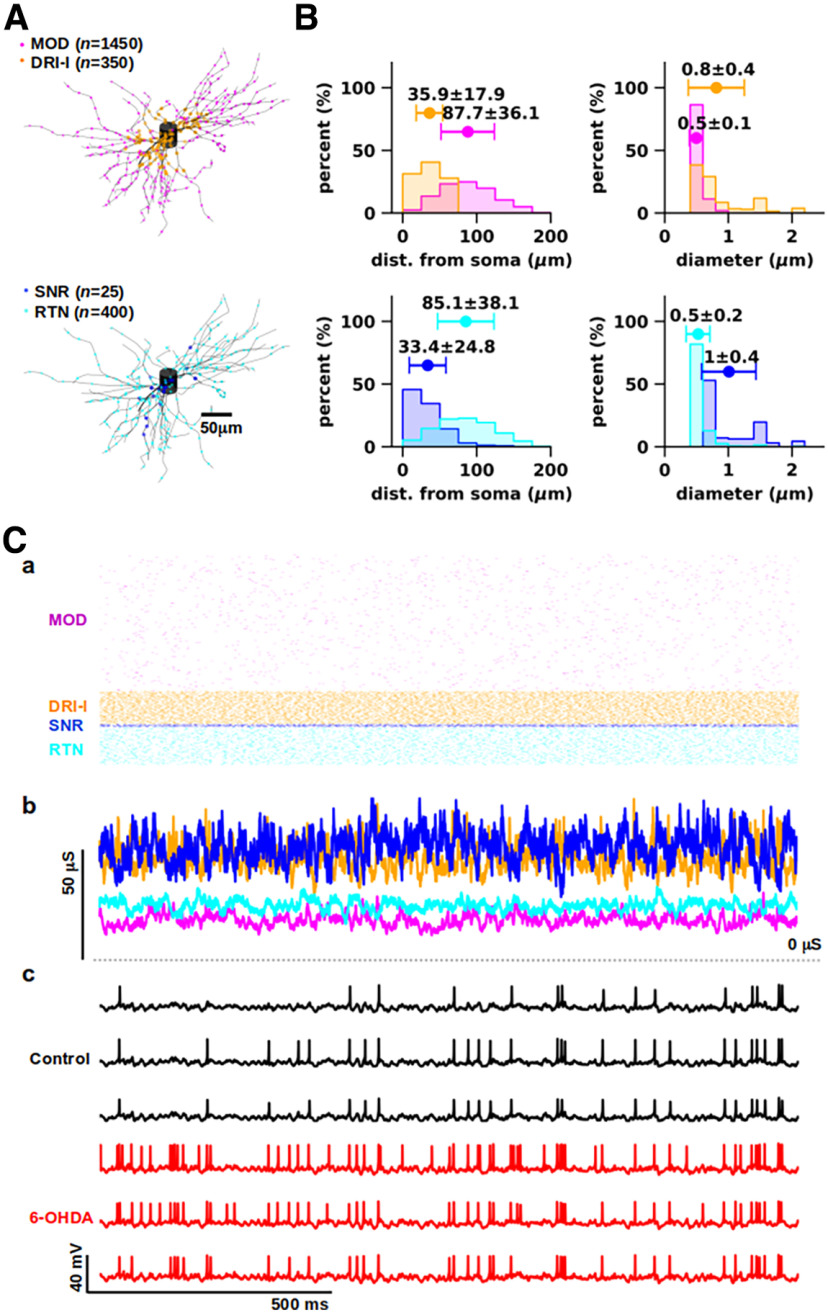
Simulation of normal and parkinsonian activity *in vivo*. ***A***, Postsynaptic locations and numbers (between parentheses) of synapses. Top, Locations of excitatory modulators (MOD; magenta) and driver-like synapses (DRI-l; orange). Bottom, Locations of inhibitory synapses from substantia nigra reticulata (SNR; blue) and reticular thalamic nuclei (RTN; cyan). ***B***, Statistical distributions of distance from soma (left) and diameters (right) of the postsynaptic dendritic segment for excitatory (top) and inhibitory (bottom) inputs (as in ***A***). The labels indicate mean ± SD of each distribution, whereas the whiskers depict the standard deviation. ***C***, Simulation of *in vivo*-like conditions. ***a***, Representative artificial spike trains representing the spontaneous presynaptic activity with physiological values of firing rate (MOD: 1.1 ± 0.1 Hz; DRI-l: 31.5 ± 0.5 Hz; SNR: 52.3 ± 0.7 Hz; RTN: 10.5 ± 0.3 Hz; ±SD) and coefficient of variation of interspike intervals (MOD: 0.43 ± 0.07 Hz; DRI-l: 0.45 ± 0.01 Hz; SNR: 0.45 ± 0.01 Hz; RTN: 0.45 ± 0.02 Hz; ±SD). ***b***, Total synaptic conductance for each set of synaptic inputs, as shown in ***a***. ***c***, Representative responses evoked by synaptic inputs to models of thalamocortical neurons in normal (black) and parkinsonian (red) states. The two states underlie the generation of different firing activity patterns.

**Figure 4.**
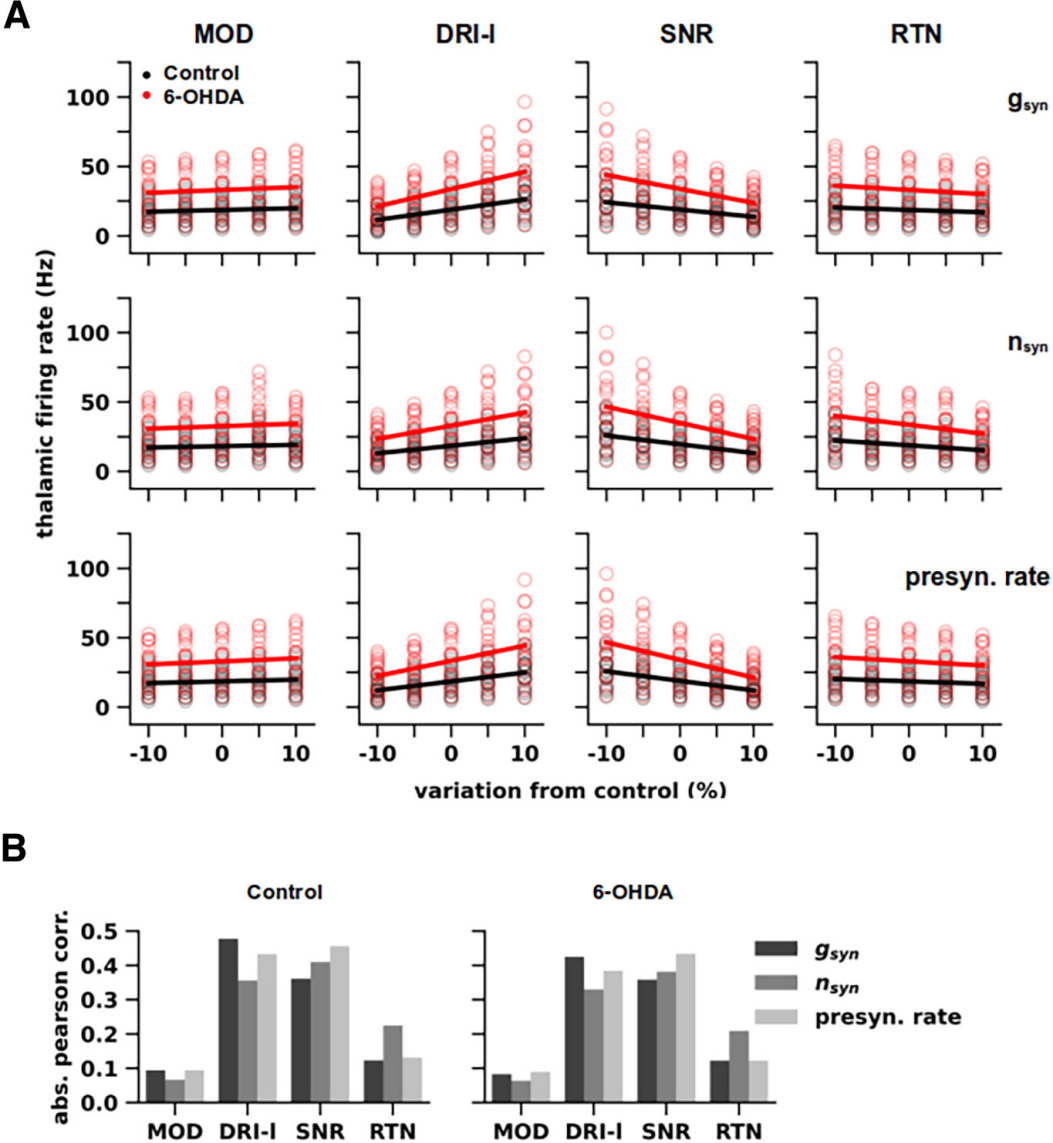
Sensitivity analysis of synaptic inputs. ***A***, Dependence of the firing rate of thalamocortical (TC) neurons in normal (black) and parkinsonian (red) states on parameter variations (±10% from control) for synaptic inputs, i.e., individual synaptic conductance (g_syn_), number of synapses (n_syn_), and presynaptic firing rate, for modulators (MOD), driver-like inputs (DRI-l), substantia nigra reticulata (SNR), and reticular thalamic nuclei (RTN). Lines were fitted on pooled responses of different TC neuron models (Control: *n* = 16; 6-OHDA: *n* = 17) obtained with multiple simulations per model (*n* =* *10). ***B***, Linear (Pearson) correlation of firing rates with the percentages of variation from control for each parameter indicated in ***A***, generated by TC neuron models in normal (left) and parkinsonian (right) states. Variations in the parameters of DRI-l and SNR inputs yielded the most significant linear correlations (Kowalski test, *p* < 0.001).

**Figure 5.**
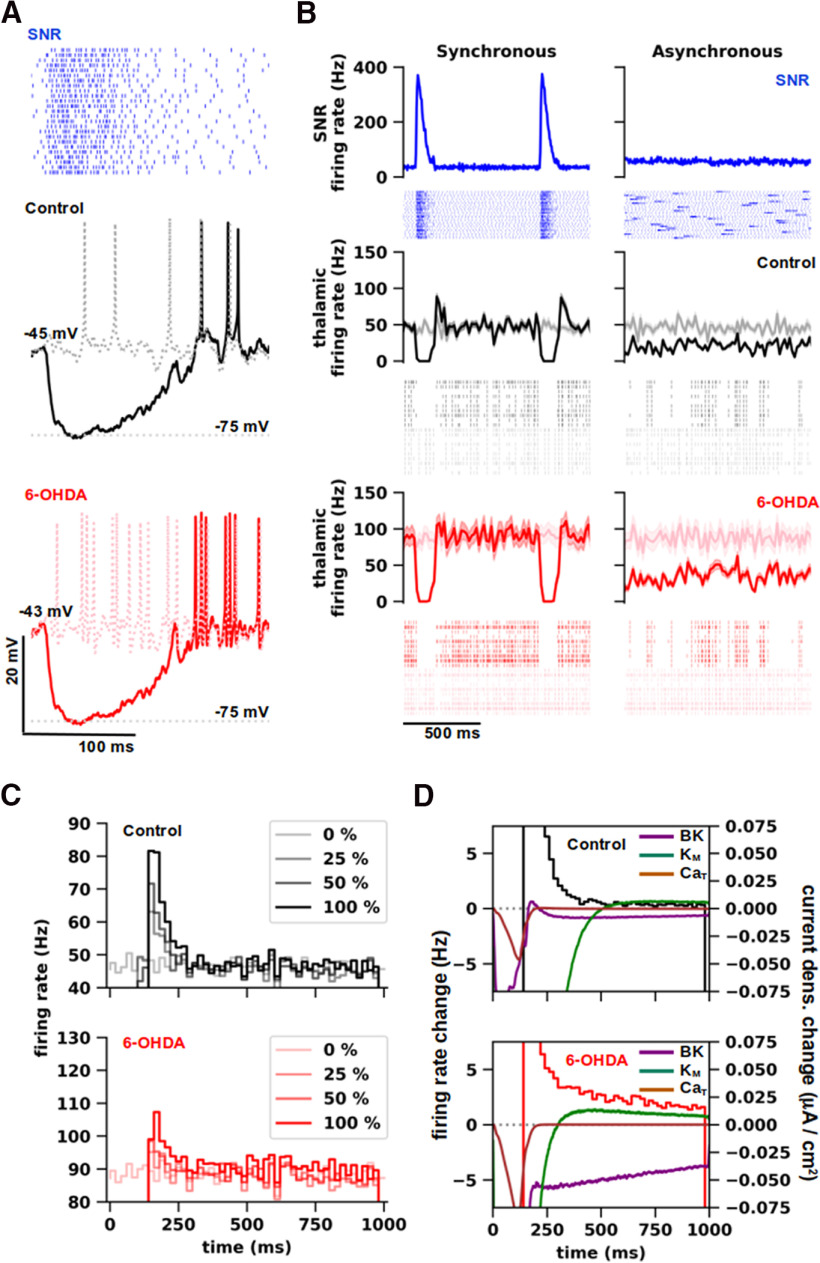
Synchronous bursting in substantia nigra reticulata evokes rebound activity in motor thalamocortical neurons. ***A***, Representative responses of thalamocortical (TC) neuron models in normal (Control; middle, black) and parkinsonian (6-OHDA; bottom, red) states to inputs from substantia nigra reticulata (SNR) with synchronous bursting (top, blue). The gray and light red dashed traces correspond to the responses in normal (top) and parkinsonian (bottom) states, respectively, without bursting in SNR (not shown). ***B***, Responses of TC neuron models in normal (black) and parkinsonian (red) states to SNR inputs (blue) with synchronous (left) and asynchronous (right) bursting. Top, Spike histograms of the presynaptic activity from SNR inputs (*n* = 25) for 10 simulations with synchronous (left) and asynchronous (right) bursting. Below the spike histograms, exemplificative raster plots of the presynaptic activity in SNR inputs (*n* = 25) for a single simulation. Each simulation was associated with a random seed that determined the subcellular distributions and activation timing (i.e., artificial spike trains) of synapses (for details, see Materials and Methods). SNR bursting was ∼150 ms in duration, with an average intraburst rate of ∼170 Hz, and an interburst rate of 35 Hz, yielding a firing rate of 55.7 ± 1.6 Hz (±SD) with a coefficient of variation of interspike interval of 0.69 ± 0.02. Middle, Bottom, Spike histograms of the pooled activity for TC neuron models in normal (*n* = 16; Control; black) and parkinsonian (*n* = 17; 6-OHDA; red) states, obtained with multiple simulations per model (*n* = 10), in presence of SNR bursting. The histograms show the instantaneous firing rate versus time for TC neuron models, with the shaded areas depicting the standard deviation. Below the spike histograms, exemplificative raster plots showing the activity of TC neuron models (*n* = 10) in normal (black, gray) and parkinsonian (red, light red) states, obtained with a single simulation, with (black, red) or without (gray, light red) bursting in SNR. ***C***, Poststimulus spike time histograms (PSTH) of TC neuron activity in normal (Control; top, red) and parkinsonian (6-OHDA; bottom, red) conditions, with different percentages of SNR inputs generating synchronous bursting (100%, 50%, 25%, 0%). The PSTHs were averaged for different models (Control: *n* = 16; 6-OHDA: *n* = 9) and multiple simulations per model (*n* = 10), over a time window of 1000 ms (1 s) in length, starting from the SNR bursting onset. ***D***, Changes in density of BK potassium (purple), M-type potassium (green), and T-type Ca^2+^ (brown) currents during postinhibitory firing activity in normal (Control; top) and parkinsonian (6-OHDA; bottom) models of TC neurons with 100% of SNR inputs generating synchronous bursting (same simulations as shown in ***C***). For each current, the changes were calculated as the difference between the curves obtained with 100% and 0% of SNR inputs generating synchronous bursting, averaged over different models (Control: *n* = 16; 6-OHDA: *n* = 17) and multiple simulations per model (*n* =* *10). The background of each panel shows the difference between PSTHs obtained with 100% and 0% of synchronous SNR bursting for normal (top) and parkinsonian (bottom) models (shown in ***C***).

**Figure 6.**
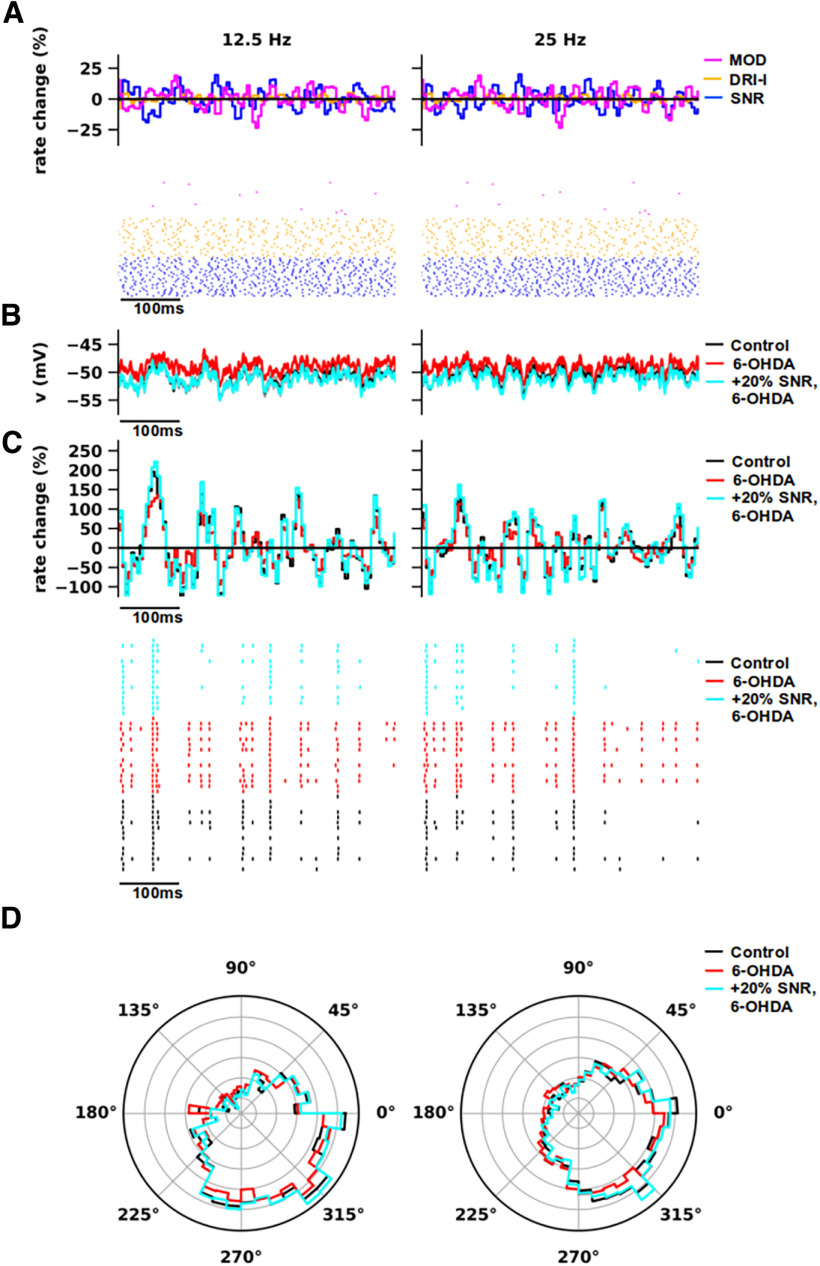
β Modulation of inhibitory inputs from substantia nigra reticulata. β Modulation in the activity of substantia nigra reticulata (SNR) at 12.5 Hz (left) and 25 Hz (right). The other synaptic inputs, such as modulators (MOD), driver-like inputs (DRI-l), and the reticular thalamic nuclei were not modulated. The spike-phase locking induced in thalamocortical (TC) neuron models by individual modulation of each synaptic input is shown in Extended Data Figure 6-1. ***A***, Spike histograms show the relative variation of instantaneous firing rate to average versus time (top) and exemplificative raster plots (bottom) of the presynaptic activity for MOD (magenta), DRI-l (orange), and SNR (blue). The spike histograms were calculated from multiple simulations (*n* = 10). The raster plots of each input were a subset of total (*n* =* *25). ***B***, Somatic voltage traces of TC neuron models in normal (Control; black) and parkinsonian (6-OHDA) states, with regular (red) and increased (+20%; cyan) SNR conductance for parkinsonian models. Each curve was obtained by averaging the voltage traces generated by models (Control: *n* = 16; 6-OHDA: *n* = 17) and multiple simulations per model (*n* = 10). ***C***, Spike histograms showing the relative variation of instantaneous firing rate to average versus time (top) and exemplificative rastergrams (bottom) of TC neuron activity in normal (Control; black) and parkinsonian (6-OHDA) states, with regular (red) and increased (+20%; cyan) SNR conductance for parkinsonian models. The spike histograms were calculated from the pooled responses of multiple models (Control: *n* = 16; 6-OHDA: *n* = 17) and multiple simulations per model (*n* = 10). Each trial was associated with a random seed that determines the subcellular distributions and activation timing (i.e., artificial spike trains) of synapses (for details, see Materials and Methods). The rastergrams show the activity of TC neuron models (*n* = 15) in normal (black) and parkinsonian states (red, cyan), obtained with a single simulation. ***D***, Phase plots of the spiking activity shown in *C* (spike histograms) for normal and parkinsonian models of TC neurons (Rayleigh, *p* < 0.001).

**Figure 7.**
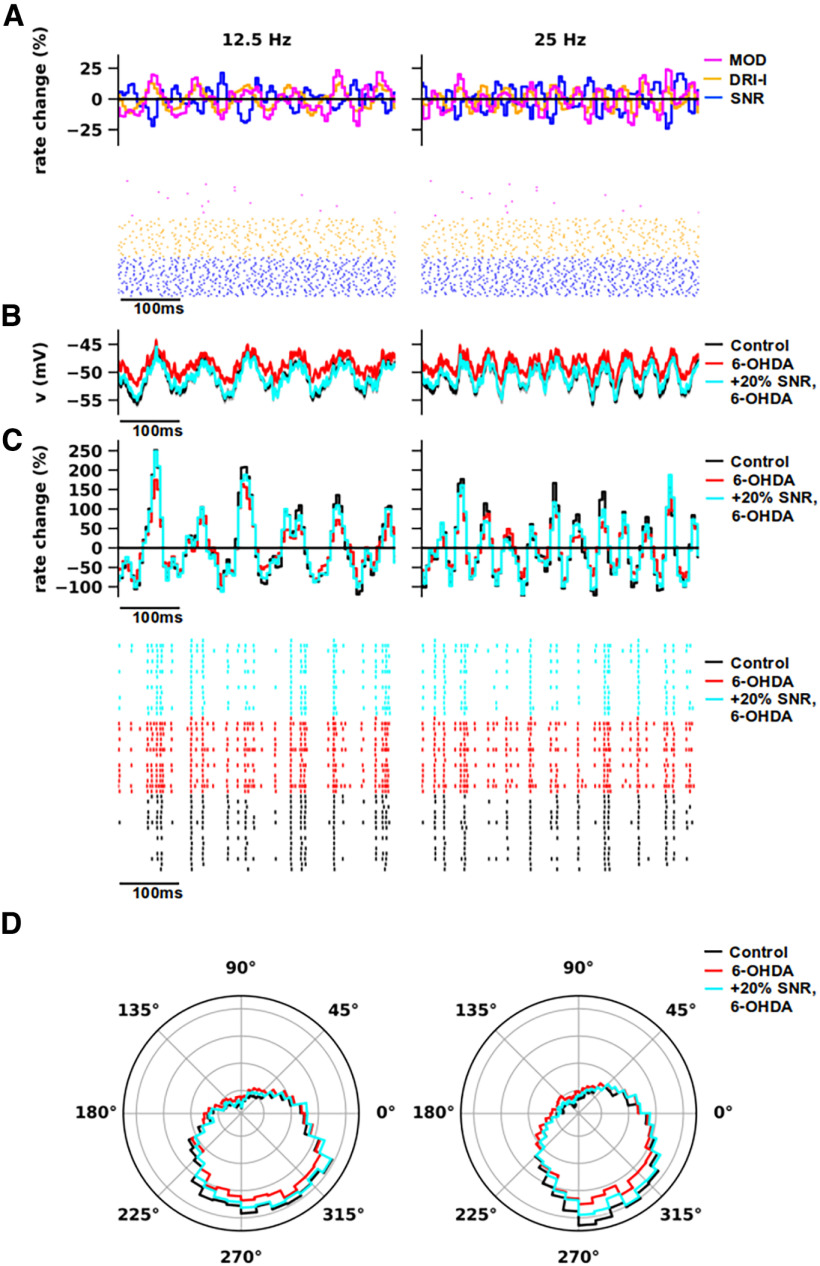
β Modulation of inhibitory inputs from substantia nigra reticulata, excitatory modulators, and driver-like inputs. β Modulation in the activity of substantia nigra reticulata (SNR), modulators (MOD), and driver-like inputs (DRI-l) at 12.5 Hz (left) and 25 Hz (right). The oscillations in MOD and DRI-l were shifted by 180° with respect to SNR inputs. The same analysis was repeated with β modulation in MOD (see Extended Data Fig. 7-1) and DRI-l (see Extended Data Fig. 7-2) inputs individually, along with SNR. Another set of simulations show the effects of adding β modulation to reticular thalamic nuclei (see Extended Data Fig. 7-3). ***A***, Spike histograms show the relative variation of instantaneous firing rate to average versus time (top) and exemplificative raster plots (bottom) of the presynaptic activity for MOD (magenta), DRI-l (orange), and SNR (blue). The spike histograms were calculated from multiple simulations (*n* = 10). The raster plots of each input were a subset of total (*n* =* *25). ***B***, Somatic voltage traces of thalamocortical (TC) neuron models in normal (Control; black) and parkinsonian (6-OHDA) states, with regular (red) and increased (+20%; cyan) SNR conductance for parkinsonian models. Each curve was obtained by averaging the voltage traces generated by models (Control: *n* = 16; 6-OHDA: *n* = 17) and multiple simulations per model (*n* = 10). ***C***, Spike histograms showing the relative variation of instantaneous firing rate to average versus time (top) and exemplificative rastergrams (bottom) of TC neuron activity in normal (Control; black) and parkinsonian (6-OHDA) states, with regular (red) and increased (+20%; cyan) SNR conductance for parkinsonian models. The spike histograms were calculated from the pooled responses of multiple models (Control: *n* = 16; 6-OHDA: *n* = 17) and multiple simulations per model (*n* = 10). Each trial was associated with a random seed that determines the subcellular distributions and activation timing (i.e., artificial spike trains) of synapses (for details, see Materials and Methods). The rastergrams show the activity of TC neuron models (*n* = 15) in normal (black) and parkinsonian states (red, cyan), obtained with a single simulation. ***D***, Phase plots of the spiking activity shown in ***C*** (spike histograms) for normal and parkinsonian models of TC neurons (Rayleigh, *p* < 0.001).

**Figure 8.**
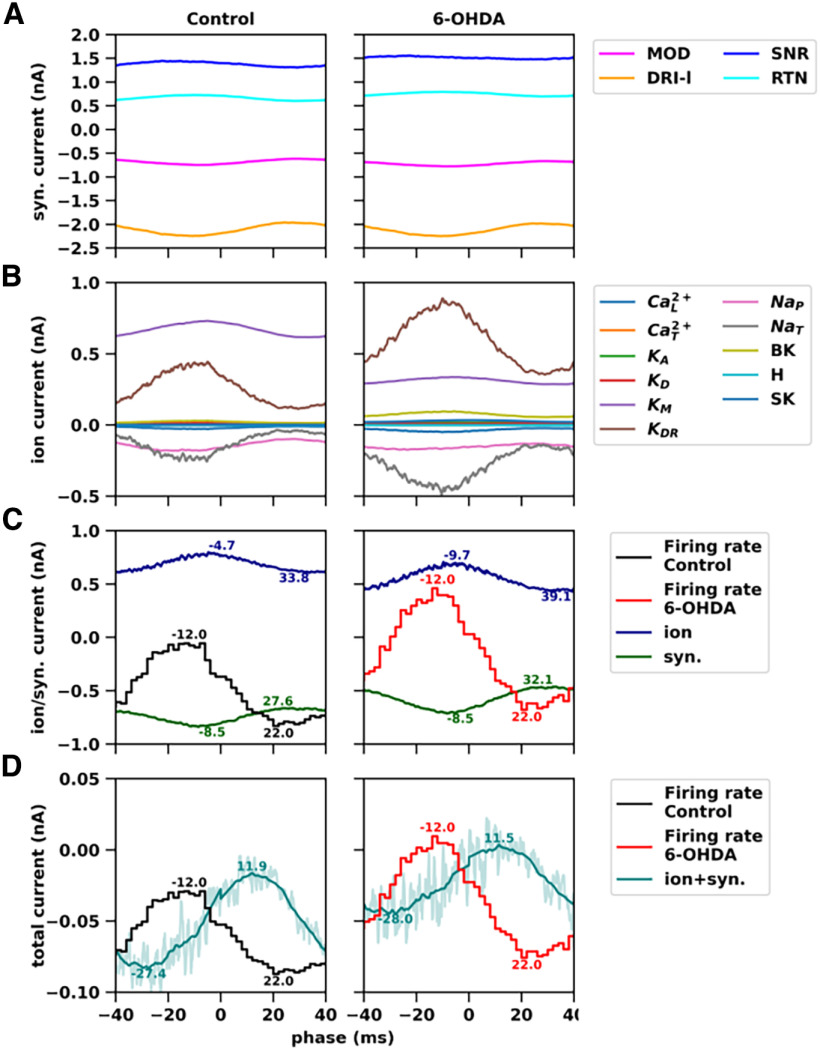
Synaptic and membrane currents resulting from β modulation of synaptic inputs at 12.5 Hz. β Modulation introduced fluctuations in the firing activity of substantia nigra reticulata (SNR), modulators (MOD), and driver-like inputs (DRI-l) at 12.5 Hz, while no β modulation occurred in the reticular thalamic nuclei (RTN). This configuration is also shown in Figure 7, left. The same analysis was performed with β modulation at 25 Hz, as shown in Extended Data Figure 8-1 (same configuration as shown in Fig. 7, right). ***A***, Total inward and outward synaptic current versus oscillation phase, calculated for each group of synaptic inputs, i.e., MOD (magenta), DRI-l (orange), SNR (blue), and RTN (cyan) inputs, in normal (left) and parkinsonian (right) states. Curves show the average current over different models (Control: *n* = 16; 6-OHDA: 17) and multiple simulations per model (*n* = 10), within a period of 80 ms, corresponding to a modulation frequency of 12.5 Hz. ***B***, Total inward (negative, depolarizing) and outward (positive, hyperpolarizing) membrane currents versus oscillation phase, calculated for each ion channel in thalamocortical (TC) neuron models in normal (left) and parkinsonian (right) conditions. The curves show the average current over different models (Control: *n* = 16; 6-OHDA: *n* = 17) and multiple simulations per model (*n* = 10), within a period of 80 ms, corresponding to a modulation frequency of 12.5 Hz. ***C***, Net synaptic current (green; individual currents shown in ***A***) and net membrane current (blue; individual currents shown in ***B***) versus oscillation phase for TC neuron models in normal (left) and parkinsonian (right) states. On the background, the curves of instantaneous firing rate versus oscillation phase for normal (left; black) and parkinsonian (right; red) TC neuron models. Labels indicate the phase of the up and down peaks for membrane and synaptic currents as well as firing rate. ***D***, Sum of the net membrane and net synaptic currents (teal; net membrane and net synaptic current are shown separately in ***C***) versus oscillation phase for TC neuron models in normal (left) and parkinsonian (right) states. On the background, the curves of instantaneous firing rate versus oscillation phase for normal (left; black) and parkinsonian (right; red) TC neuron models. Labels indicate the phases of the up and down peaks for the sum of the net currents as well as firing rate.

To represent presynaptic activity, we generated artificial spike trains (Abbasi et al., 2020), with firing rates consistent with experimental estimates (DRI-l: 30 Hz; MOD: 1.1 Hz; RTN: 10 Hz; SNR: 50 Hz; Sirota et al., 2005; Huh and Cho, 2016; Barrientos et al., 2019; Inagaki et al., 2022). Specifically, we used an algorithm that generates a random sequence of interspike intervals picked from a γ distribution with a refractory period and “shape” parameter set a priori (Abbasi et al., 2020). We targeted generic spike properties with a coefficient of variation of interspike intervals (CV_ISI_) of 0.45 (as observed in SNR during *in vivo* recordings; Lobb and Jaeger, 2015), obtained with a shape of 5 ($CV_{ISI}=\frac{1}{\sqrt{shape}}$), and a refractory period of 3 ms. Unless otherwise noted, we maintained these combinations of parameters for all the synaptic inputs (Figs. 3-8).

10.1523/ENEURO.0237-23.2023.tab3-1 Extended Data Table 3-1 Equations of active membrane properties. Bold literals indicate the free parameters (see Table 3). Download Figure 3-1, TIF file.

10.1523/ENEURO.0237-23.2023.f6-1 Extended Data Figure 6-1 β Modulation of different synaptic inputs individually. Related to Figure 6. Phase plots of the spiking activity for normal (black) and parkinsonian (red) models of thalamocortical (TC) neurons in presence of β modulation in the firing activity of substantia nigra reticulata (SNR), excitatory modulators (MOD), excitatory driver-like (DRI-l) inputs, and reticular thalamic nuclei (RTN). For each group of synaptic inputs, β modulation induces significant spike-phase locking in the activity of TC neuron models in both states (Rayleigh, *p* < 0.001). Each phase plot was obtained by averaging the responses of different TC neuron models (Control: *n* = 16; 6-OHDA: *n* = 17) and multiple simulations per model (*n* = 10). Compared to MOD and RTN inputs (128.1–154.9°), the circular SD of the spiking activity in TC neuron models achieved the lowest values with β modulation in SNR and DRI-l inputs (93.4–107.4°), suggesting that SNR and DRI-l inputs can induce the strongest spike-phase locking in TC neurons. Download Figure 6-1, TIF file.

10.1523/ENEURO.0237-23.2023.f7-1 Extended Data Figure 7-1 β Modulation of inhibitory inputs from substantia nigra reticulata and excitatory driver-like inputs. β Modulation in the activity of substantia nigra reticulata (SNR) and driver-like inputs (DRI-l) at 12.5 Hz (left) and 25 Hz (right). Modulators (MOD) were not modulated. The oscillations in DRI-l were shifted by 180° with respect to SNR inputs. The effects of β modulation in SNR only are shown in Figure 7. ***A***, Spike histograms show the relative variation of instantaneous firing rate to average versus time (top) and exemplificative raster plots (bottom) of the presynaptic activity for MOD (magenta), DRI-l (orange), and SNR (blue). The spike histograms were calculated from multiple simulations (*n* = 10). The raster plots of each input were a subset of total (*n* =* *25). ***B***, Somatic voltage traces of thalamocortical (TC) neuron models in normal (Control; black) and parkinsonian (6-OHDA) states, with regular (red) and increased (+20%; cyan) SNR conductance for parkinsonian models. Each curve was obtained by averaging the voltage traces generated by models (Control: *n* = 16; 6-OHDA: *n* = 17) and multiple simulations per model (*n* = 10). ***C***, Spike histograms showing the relative variation of instantaneous firing rate to average versus time (top) and exemplificative rastergrams (bottom) of TC neuron activity in normal (Control; black) and parkinsonian (6-OHDA) states, with regular (red) and increased (+20%; cyan) SNR conductance for parkinsonian models. The spike histograms were calculated from the pooled responses of multiple models (Control: *n* = 16; 6-OHDA: *n* = 17) and multiple simulations per model (*n* = 10). Each trial was associated with a random seed that determines the subcellular distributions and activation timing (i.e., artificial spike trains) of synapses (for details, see Materials and Methods). The rastergrams show the activity of TC neuron models (*n* = 15) in normal (black) and parkinsonian states (red, cyan), obtained with a single simulation. ***D***, Phase plots of the spiking activity shown in ***C*** (spike histograms) for normal and parkinsonian models of TC neurons (Rayleigh, *p* < 0.001). Download Figure 7-1, TIF file.

10.1523/ENEURO.0237-23.2023.f7-2 Extended Data Figure 7-2 β Modulation of inhibitory inputs from substantia nigra reticulata and excitatory modulators. β Modulation in the activity of substantia nigra reticulata (SNR) and excitatory modulators (MOD) at 12.5 Hz (left) and 25 Hz (right). Driver-like inputs (DRI-l) were not modulated. The oscillations in MOD were shifted by 180° with respect to SNR inputs. The effects of β modulation in SNR only are shown in Figure 7. ***A***, Spike histograms show the relative variation of instantaneous firing rate to average versus time (top) and exemplificative raster plots (bottom) of the presynaptic activity for MOD (magenta), DRI-l (orange), and SNR (blue). The spike histograms were calculated from multiple simulations (*n* = 10). The raster plots of each input were a subset of total (*n* =* *25). ***B***, Somatic voltage traces of thalamocortical (TC) neuron models in normal (Control; black) and parkinsonian (6-OHDA) states, with regular (red) and increased (+20%; cyan) SNR conductance for parkinsonian models. Each curve was obtained by averaging the voltage traces generated by models (Control: *n* = 16; 6-OHDA: *n* = 17) and multiple simulations per model (*n* = 10). ***C***, Spike histograms showing the relative variation of instantaneous firing rate to average versus time (top) and exemplificative rastergrams (bottom) of TC neuron activity in normal (Control; black) and parkinsonian (6-OHDA) states, with regular (red) and increased (+20%; cyan) SNR conductance for parkinsonian models. The spike histograms were calculated from the pooled responses of multiple models (Control: *n* = 16; 6-OHDA: *n* = 17) and multiple simulations per model (*n* = 10). Each trial was associated with a random seed that determines the subcellular distributions and activation timing (i.e., artificial spike trains) of synapses (for details, see Materials and Methods). The rastergrams show the activity of TC neuron models (*n* = 15) in normal (black) and parkinsonian states (red, cyan), obtained with a single simulation. ***D***, Phase plots of the spiking activity shown in ***C*** (spike histograms) for normal and parkinsonian models of TC neurons (Rayleigh, *p* < 0.001). Download Figure 7-2, TIF file.

10.1523/ENEURO.0237-23.2023.f7-3 Extended Data Figure 7-3 β Modulation of inhibitory inputs from substantia nigra reticulata, excitatory modulators and driver-like inputs, and reticular thalamic nuclei. ***A***, Phase plots of the spiking activity shown in Figure 7 for normal (black) and parkinsonian (red) models of thalamocortical neurons (Rayleigh, *p* < 0.001). The spike histograms were calculated from the pooled responses of different models (Control: *n* = 16; 6-OHDA: *n* = 17) and multiple simulations per model (*n* = 10). ***B***, Same as in ***A*** with β modulation of reticular inputs in phase with inputs from substantia nigra reticulata (SNR; Rayleigh, *p* < 0.001). ***C***, Same as in ***A*** with β modulation of reticular inputs shifted by 180° with respect to SNR inputs (Rayleigh, *p* < 0.001). Download Figure 7-3, TIF file.

10.1523/ENEURO.0237-23.2023.f8-1 Extended Data Figure 8-1 Synaptic and membrane currents resulting from β modulation of synaptic inputs at 25 Hz. Same simulations as shown in Figure 8 with β modulation at 25 Hz, using the same configuration of inputs shown also in Figure 7, right. Download Figure 8-1, TIF file.

We enhanced the published method to generate bursts, treated as a special case of event in each spike train. Specifically, each burst sequence was generated separately and subsequently merged with an existing baseline firing spike train. A template described the time course of the intraburst firing rate, which can be manipulated to fit different accelerating or decelerating patterns. Specifically, we used different values of shape for the γ distributions of interspike intervals for regular spike trains (=5) and bursts (=3), which yielded bell shaped and more symmetric distributions for the former, and highly right skewed ones for the latter (Selinger et al., 2007). Furthermore, interburst intervals were governed by a γ distribution, associated with mean and regularity parameters that correlates with average (positively) and variance (negatively) of the interburst intervals, respectively.

We then generated bursting activity for SNR inputs (Fig. 5), as artificial spike trains of ∼150 ms in duration, with an average firing rate of ∼170 Hz and a refractory period of 1.5 ms. For the interburst activity, we decreased the baseline firing rate to 35 Hz and increased the regularity to 50. The interburst intervals were 1 s on average, with a minimum value of 150 ms and regularity values of 5 and 50,000 for spike trains with asynchronous and synchronous bursting, respectively. Overall, this configuration yielded artificial spike trains with firing rate of 55.7 ± 1.6 Hz (±SD) and coefficient of variation of interspike interval of 0.69 ± 0.02 (±SD), consistent with previous experimental recordings from SNR (Lobb and Jaeger, 2015).

### Data analysis

We used a custom version of the eFEL python package for feature extraction from membrane potential traces (https://efel.readthedocs.io). The junction potential of the intracellular medium was estimated using JPcalc software (Barry, 1994). For each simulated experiment, reversal potentials of ion species were estimated by using the Nernst equation, accounting for the composition of the aCSF (see Table 1). For statistical analyses, we used Python3 with the SciPy and NumPy packages. To compare f-I curves (Fig. 2), we used the Wilcoxon matched-pairs signed rank test. To compare different sets of measures, we used Mann–Whitney *U* tests. To assess the significance of the Pearson correlation (Fig. 3), we used the Kowalski test. To assess the significance of spike-phase locking (Figs. 6, 7), we used the Rayleigh test on polar data. The confidence intervals were presented as mean ± SD, mean ± standard SE. We preferred the SD for the estimates of firing rates, the coefficient of variations for the interspike intervals, and the distributions of distance from soma and diameters of the postsynaptic dendrites (Fig. 3), to provide an immediate measure of the spread around the mean, while we used the SE in all the other cases. To smooth the curves of net current shown in Figure 8 and Extended Data Figure 8-1*D*, we applied a first-order filtering of Savitzky–Golay with a kernel of 101 points in size.

## Results

The effects of unilateral 6-OHDA lesions on VM neural properties were previously recorded *in vitro* in mice (Bichler et al., 2021). The results showed that the excitability of TC neurons was increased in the dopamine-depleted hemisphere. This effect was attributed to M-type potassium current suppression. To understand how these excitability changes may impact synaptic integration *in vivo*, we constructed biophysically detailed VM TC neuron models in normal and 6-OHDA depleted conditions (Fig. 1; for details, see Materials and Methods, Fitting neuron models), using the NEURON simulator (Hines and Carnevale, 1997). We modeled the main afferent inputs to VM, i.e., excitatory modulators (MOD), excitatory driver-like inputs (DRI-l), inhibitory inputs from substantia nigra reticulata (SNR), and inhibitory inputs from reticular thalamic nuclei (RTN), to simulate *in vivo*-like conditions (Fig. 1; for details, see Simulation of *in vivo*-like conditions). This approach allowed us to assess the interaction of intrinsic thalamic neural dynamics with synaptic input properties expected in normal or parkinsonian waking conditions through measurements in the model that cannot be performed experimentally *in vivo*.

### Simulation of *in vitro* experiments

Physiologically, VM TC neurons display the characteristic firing modes, e.g., tonic firing, low-threshold spiking, and rebound bursting (Edgerton and Jaeger, 2014; Bichler et al., 2021), commonly observed in other thalamic nuclei (Llinás and Steriade, 2006), while dopamine depletion increases tonic firing and prolongs rebound bursting in these neurons (Bichler et al., 2021). Here, we tested the ability of the VM TC neuron models in reproducing *in vitro* recordings and the changes following dopamine depletion, comparing simulation and experimental responses.

Specifically, we considered whole-cell recordings from slices of adult mice VM in normal conditions and after unilateral 6-OHDA application (Control: *n* = 9; 6-OHDA: *n* = 7), stimulated by depolarizing (20–300 pA; 2 s of duration; Fig. 2*A*,*B*) or hyperpolarizing (from –50 pA to –200 pA; 2 s of duration; Fig. 2*C*,*D*) current steps on top of a bias current (Control: 93.3 ± 18.7; 6-OHDA: 67.1 ± 12.7; ±SE). In particular, the bias current held the membrane potential to –69 mV, inactivating T-type Ca^2+^ channels, and thus enabled the generation of tonic firing in response to depolarizing steps (Fig. 2*A*, left), while hyperpolarizing steps evoked rebound bursting (Fig. 2*C*, left). 6-OHDA enhanced the excitability of TC neurons, increasing responses of TC neurons in tonic firing (Fig. 2*A*, left; compare black and red traces), assessed as a shift of the f-I curves to the left (Wilcoxon, *p* < 0.01; Fig. 2*B*, left; compare black and red curves), and prolonged rebound bursting (Fig. 2*A*, left, compare black and red traces), assessed as an increase in the rebound spike count (Mann–Whitney, 20–40 mV: *p* = 0.012; 40–60 mV: *p* < 0.01; Fig. 2*D*, left, compare black and red bars for each bracket).

To allow a direct comparison between the two sets of traces, the simulations reproduced the stimulation protocols (e.g., step and bias current amplitudes) and the environmental conditions of slices (e.g., temperature, reversal potential of ion species, holding membrane potential; Table 1). We thus observed that the models (Control: *n* = 16; 6-OHDA: *n* = 17) replicated the responses in tonic and burst firing modes observed experimentally, in response to depolarizing and hyperpolarizing steps, respectively, with realistic levels of firing rate adaptation and action potential properties (e.g., AP height, AHP depth, firing rate adaption). Both experimental and simulation traces displayed variability in multiple firing properties (Extended Data Figs. 2-1 and 2-2). Compared with normal models, parkinsonian models displayed stronger excitability, generating more action potentials in response to depolarizing and hyperpolarizing current steps (Fig. 2*A*,*C*, right, compare black and red traces). Parkinsonian models generated significantly higher spike rates throughout the entire range of stimulation using depolarizing currents (Wilcoxon, *p* < 0.01; Fig. 2*B*, right, compare black and red curves), while the spike rates were comparable to the values observed experimentally for both normal and parkinsonian models (Fig. 2*B*, compare experimental and simulation f-I curves in left and right panels, respectively). Likewise, rebound spike counts were statistically different in normal and parkinsonian models (Mann–Whitney, 20–40 mV: *p* < 0.01; 40–60 mV: *p* < 0.01; Fig. 2*D*, compare black and red bars for each bracket) but comparable to the corresponding experimental values (Fig. 2*D*, compare experimental and simulation spike counts for each bracket in left and right panels, respectively).

The models discussed above resulted from a selection process comprising several quality checks. One check concerned the ability of the models in replicating the effects of XE-991 application (10–20 μm; Bichler et al., 2021), simulated by reducing the M-type conductance density by 70% (Yeung and Greenwood, 2005), which shifted the f-I curves to the left significantly for normal but not parkinsonian models (not shown), in accord with our findings *in vitro* (Bichler et al., 2021). This yielded parkinsonian models with significantly lower expression of M-type potassium current than control (Mann–Whitney, *p* < 0.01; Control: 306.76 ± 29.24 μS/cm^2^; 6-OHDA: 9.69 ± 2.46 μS/cm^2^; ±SE; Table 3). Increasing M-type potassium current in parkinsonian models by ∼40 times rescued the effects of dopamine depletion (not shown), making the f-I curves of parkinsonian models indistinguishable from control (Wilcoxon, *p* = 0.30), and reducing their rebound spike count to similar values as observed for control (Mann–Whitney, 20–40 mV: *p* = 0.026; 40–60 mV: *p* = 0.013). Taken together, these simulations demonstrated that our models replicate the firing behaviors of VM TC neurons observed *in vitro* along with the alterations caused by dopamine depletion.

### Simulation of *in vivo*-like conditions

After fitting the TC neuron models to *in vitro* recordings, we modeled the main afferent inputs to VM (Fig. 3): (1) modulators (MOD), approximating the glutamatergic inputs conveyed from layer 6 of MC; (2) driver-like inputs (DRI-l), approximating glutamatergic inputs from layer 5 of MC and motor-related subcortical areas; and GABAergic inputs from (3) substantia nigra reticulata (SNR) and (4) reticular thalamic nucleus (RTN).

For each group of synapses, we constrained synaptic physiology, subcellular distributions, number of terminals, and individual conductance to experimental data (for details, see Materials and Methods, Modeling synaptic inputs). In particular, the distribution of DRI-l terminals reproduced the distance from soma observed for deep cerebellar nuclei terminals in VM (Fig. 3*A*,*B*; Gornati et al., 2018), while the distributions for MOD, RTN, and SNR were modeled on our electron microscopy data (Fig. 3*A*,*B*; for details, see Materials and Methods, Modeling synaptic inputs). As an emergent property, we obtained a co-localization along the somato-dendritic arbor of VM TC neuron models (Fig. 3*B*) for DRI-l and SNR terminals, targeting proximal dendrites with larger diameters, as well as MOD and RTN terminals, targeting distal dendrites with small diameters, suggesting that a stronger interaction takes place in VM TC neurons between each pair of co-localized terminals.

We then tested the impact of these synaptic inputs on normal and parkinsonian TC neuron models (Fig. 3*C*). We represented the presynaptic activity with artificial spike trains replicating firing rates and irregularity observed experimentally (Fig. 3*Ca*; for details, see Materials and Methods, Modeling synaptic inputs). With these configurations of synaptic inputs, we observed that the highest values of total synaptic conductance over time were achieved by SNR and DRI-l inputs (Fig. 3*Cb*), suggesting that these two classes of synaptic inputs sustained the TC neuron firing in normal and parkinsonian states (see also below, Sensitivity analysis of synaptic response function). Although the two populations of models received the same synaptic inputs, they generated different firing responses (Fig. 3*Cc*). Compared with control, parkinsonian models displayed increased firing rates (Mann–Whitney, *p* < 0.0001; Control: 18.6 ± 9.4 Hz; 6-OHDA: 33.0 ± 17.3 Hz; ±SD) and baseline voltage (Mann–Whitney, *p* < 0.0001; Control: −51.4 ± 1.2 mV; 6-OHDA: −48.4 ± 1.3 mV; ±SD). Therefore, the models suggest that the increased excitability observed *in vitro* for parkinsonian states (Bichler et al., 2021) resulted in a strong firing increase with a balance of excitatory and inhibitory synaptic inputs as well.

### Sensitivity analysis of synaptic response function

To establish the importance of each synaptic afferent, we performed a sensitivity analysis of their parameters, i.e., numbers of synapses (*n_syn_*), unitary conductance (*g_syn_*), and presynaptic firing rate. For each group of synapses, we plotted the thalamic firing rate for normal and parkinsonian models as a function of each parameter, varied by ±10% from control (Fig. 4*A*). For each parameter, we performed a linear regression between the percent of variation and the thalamic firing rate in normal and parkinsonian states (Fig. 4*A*, black and red lines), and used the absolute value of the Pearson correlation between thalamic firing rate and the varying parameter (Fig. 4*B*).

In normal and parkinsonian states, increasing synapse number, conductance, or firing rate for RTN and SNR inputs decreased firing rates of the TC neuron models (i.e., negative correlation), while increasing these parameters for MOD and DRI-l inputs resulted in the opposite effect (i.e., positive correlation; Fig. 4*A*). The fitted lines for each parameter were steeper for DRI-l and SNR inputs, whereas they appeared flat for MOD and RTN inputs (Fig. 4*A*), and the firing rate was higher in the parkinsonian state than the control state throughout the entire range of variation. By comparing the absolute values of correlation, we observed strong (0.38–0.50; Fig. 4*B*) and significant correlations for all the parameters related to DRI-l and SNR inputs (Kowalski test, *p* < 0.0001), whereas weak correlations were observed for MOD and RTN inputs (0.08–0.27; Fig. 4*B*), insignificant for all the parameters related to MOD and RTN inputs, except the number of RTN synapses, which however displayed a weaker significance than DRI-l and SNR inputs (Kowalski test, Control: *p* = 0.015; 6-OHDA: *p* = 0.014). Taken together, these simulations suggest that, under these configurations of afferent inputs, VM output *in vivo* is primarily driven by DRI-l and SNR but not MOD and RTN inputs, without any relevant difference between normal and parkinsonian states. This is consistent with the observation that synaptic inputs from SNR and DRI-l yielded the highest values of total synaptic conductance over time (Fig. 3). Additionally, synaptic inputs evoked stronger responses in parkinsonian states throughout the entire range of parameters, suggesting a robust effect of dopamine depletion on VM TC neuron excitability.

### Effects of synchronous bursting inputs from substantia nigra reticulata

Previous speculations have described thalamic rebound bursting as a key mechanism of parkinsonian pathophysiology (Rubin and Terman, 2004; Meijer et al., 2011), and experiments showed that the occurrence of rebound bursting in ventrolateral thalamus coincides with the emergence of motor deficits (Kim et al., 2017). *In vitro* experiments showed that synchronous activation of nigral axons could evoke rebound bursting in VM, where dopamine depletion enhanced rebound bursting evoked by hyperpolarizing current steps (Bichler et al., 2021). Additionally, *in vivo* experiments showed that dopamine depletion increased spike synchrony and bursting in SNR (Wang et al., 2010; Anderson et al., 2015; Willard et al., 2019), an ideal mechanism for evoking rebound spiking in VM. While these findings support the possibility that rebound spiking in VM might contribute to parkinsonian dynamics, this hypothesis has not yet been directly tested in behaving animals.

To investigate the effects of synchronous nigral bursting on VM output, we generated trains of nigral bursts reproducing the rates and duration observed in our experimental recordings in dopamine-depleted mice (for details, see Materials and Methods, Presynaptic activity: artificial spike train generation). We observed that synchronous SNR bursts hyperpolarized the membrane of TC neuron models (Fig. 5*A*,*B*), blocking firing activity in normal (black) and parkinsonian (red) states, followed by a temporary firing rate increase at the offset of the nigral bursting. Comparing the thalamic activity evoked by SNR inputs with (Fig. 5*B*, black, red) and without bursts (Fig. 5*B*, gray, light red), we observed that the postinhibitory increase in firing rate occurred robustly at the offset of the SNR bursting, without affecting the firing activity over different time windows (Fig. 5*B*, left). Instead, asynchronous SNR bursting decreased the average firing rates throughout the entire course of firing activity (Fig. 5*B*, right). By comparing the poststimulus spike time histograms with (black, red) and without (gray, light red) synchronous nigral bursting (Fig. 5*B*), we observed that SNR bursting evoked a significant postinhibitory firing rate increase in most TC neuron models (Control: 16/16; 6-OHDA: 9/17; Fig. 5*C*), with different courses in normal and parkinsonian states, reaching a peak instantaneous firing rate of ∼80 and ∼110 Hz, respectively, and lasting up to 120 and 460 ms, respectively. Both the firing rate peak and the duration of the postinhibitory firing rate increase positively correlated with the percentage of synchronous SNR inputs (Fig. 5*C*), maintaining different peaks and durations in the two states (Fig. 5*C*,*D*). Therefore, our simulations suggest that synchronous SNR bursting evokes postinhibitory spiking activity in motor thalamus with different courses in normal and parkinsonian states, and that the intensity of this effect correlates with the percentage of synchronous SNR inputs.

To seek a mechanistic explanation for the difference between the postinhibitory responses observed in normal and parkinsonian models, we analyzed the variation in transmembrane current density induced by each HH-conductance over time (Fig. 5*D*). Surprisingly, the postinhibitory increase in firing rate following synchronous SNR bursting was not sustained by T-type Ca^2+^ current, as the hyperpolarization was not sufficient to de-inactivate these channels (Fig. 5*D*, brown lines). Instead, the early increase at the offset of the hyperpolarization coincided with a state of incomplete recovery for M-type potassium current (Fig. 5*A*, green lines), observed with both normal and parkinsonian models. Additionally, with parkinsonian models only, the incomplete recovery of BK potassium current, which maintained the current lower than baseline by ∼0.03–0.07 μS/cm^2^ for ∼800 ms, contributed to both early and late parts of postinhibitory firing rate increase (Fig. 5*D*, bottom, purple line). Therefore, these predictions suggest that synchronous SNR bursting *in vivo* cannot de-inactivate T-type Ca^2+^ channels enough to evoke rebound bursting in TC neurons, while it causes a postinhibitory increase in firing activity, due to the slow recovery of M-type potassium current for models in both states, along with the slow recovery of the BK potassium current in parkinsonian models only.

### Effects of β modulation in the synaptic afferents to ventromedial thalamus

Another hallmark of parkinsonian pathophysiology is the exaggeration of β oscillations in VM, observed by local field potential (LFP) recordings (Brown et al., 2001; Cassidy et al., 2002; Levy et al., 2002; Kühn et al., 2005; Sharott et al., 2018), with increased spike-LFP coherence in VM, SNR, and MC (Brazhnik et al., 2012, 2016; Nakamura et al., 2021). Therefore, it has been hypothesized that oscillations might originate in SNR and then entrain VM, propagating globally throughout the entire motor cortico-thalamocortical loop. We tested this hypothesis by introducing fluctuations in the firing activity of SNR within the β band, i.e., at 12.5 and 25 Hz (Fig. 6*A*, blue curve and raster), with amplitude ±10% from the baseline. We observed that somatic membrane potential (Fig. 6*B*) and instantaneous firing rate (Fig. 6*C*) reflected the fluctuations in SNR, with significant spike-phase locking (Rayleigh, *p* < 0.001; Fig. 6*D*). Compared with SNR, the oscillations in firing rate of VM TC neuron models achieved higher amplitudes, with down and up phases of –90% and +148% on average, respectively, without any noticeable difference between normal and parkinsonian states, suggesting that VM does not only relay but also amplifies the β oscillations in SNR in either state.

Moreover, canonical models of PD suggest that excessive nigrothalamic inhibition, due to increased SNR firing rate, might impede thalamocortical transmission of motor signals, preventing the regular course of movements (DeLong, 1990). While our simulations suggested that increased firing rate and/or synaptic conductance of SNR inputs could be two ways of enhancing nigrothalamic inhibition (Fig. 3), studies on rodents led to inconsistent conclusions about the changes in SNR firing rate after dopamine depletion, with some experiments showing no changes or even a decrease in firing rate across different sets of SNR neurons (Díaz et al., 2003; Wang et al., 2010; Anderson et al., 2015; Lobb and Jaeger, 2015; Willard et al., 2019). Therefore, these findings suggest the possibility that enhanced SNR inhibition could be a consequence of increased synaptic conductance rather than firing rate. We tested this hypothesis by increasing unitary synaptic conductance by 20% for SNR on parkinsonian models, yielding a basal firing rate indistinguishable from control (Mann–Whitney, *p* = 0.41; 17.9 ± 10.1 vs 18.6 ± 9.4 Hz; ±SD), consistent with previous observations from basal ganglia receiving nuclei of motor thalamus (Anderson et al., 2015; Di Giovanni et al., 2020; Nakamura et al., 2021). The increase did not affect the oscillatory firing patterns in the VM (Fig. 6*B–D*, compare black, red, and cyan curves), suggesting that enhanced nigrothalamic inhibition by itself did not increase spike-phase locking.

Additionally, significant spike-phase locking was induced by β-modulating each synaptic input individually (Rayleigh, *p* < 0.001; Extended Data Fig. 6-1). To compare the levels of spike-phase locking in VM with β oscillations of each synaptic input, we calculated the circular standard deviation of spike times. In this analysis, the stronger spike-phase locking is revealed by a lower circular standard deviation of spike times. We thus observed that the circular standard deviation was lower with β modulation of SNR and DRI-l inputs (93.4−107.4°) than MOD and RTN inputs (128.1−154.9°), suggesting that β oscillations in VM are primarily conveyed from SNR and DRI-l inputs.

LFP recordings revealed that dopamine depletion increased β oscillations in layer 5/6 of MC (Brazhnik et al., 2012) and the spike-phase locking within SNR (Brazhnik et al., 2012; Nakamura et al., 2021). To study how these oscillations could entrain VM, we added β modulation in MOD and DRI-l synapses, approximating glutamatergic inputs to VM from layer 6 and 5 of MC in our simulations, respectively. Their phases were shifted by 180° with respect to SNR, consistent with previous experimental recordings (Brazhnik et al., 2012), which prevented a cancellation of inhibitory and excitatory input modulation. Adding β modulation in both the MOD and DRI-l inputs (Fig. 7*A*) yielded subthreshold oscillations in somatic voltage traces (Fig. 7*B*) as well as instantaneous firing rates (Fig. 7*C*) and significant spike-phase locking (Rayleigh, *p* < 0.001; Fig. 7*D*). In particular, the oscillations in firing rate displayed down and up phases of –92% and +152% on average, confirming that VM amplifies the β oscillations present in SNR, MODs, and DRI-l synaptic inputs. Compared with β modulation in SNR input alone, adding β modulation in the MOD synapses only slightly enhanced the oscillatory firing activity in VM (Extended Data Fig. 7-1), decreasing the circular standard deviation of spike times by 4.4−8.2° (Table 5). Similarly, adding β modulation only to RTN inputs had a minor impact on VM firing (Extended Data Fig. 7-3). Instead, adding β modulation in only the DRI-l inputs increased both spike-phase locking and amplification in firing rate oscillations (Extended Data Fig. 7-2). This could be assessed as a decrease in the circular standard deviation of spike times by 19.1−27.9°, which was substantially stronger than observed with β modulation in SNR and MOD inputs (Table 5, compare “SNR+DRI-l” with “SNR” and “SNR+MOD”). The increased effects on VM activity observed by adding β modulation in both MOD and DRI-l inputs were comparable with those observed by adding β modulation in DRI-l inputs only (Extended Data Fig. 7-2), suggesting that cortical inputs from layer 5 were more effective than those from layer 6 in transmitting β oscillations to VM. We observed that increasing SNR conductance in parkinsonian models slightly reduced the circular standard deviations of spike times by 6.7−8.6° (Table 5). Therefore, our simulations suggest that enhanced SNR inhibition refines the transmission of β oscillations from MOD and DRI-l inputs to VM.

**Table 5:** Circular statistics of spike-phase locking in normal and parkinsonian states with oscillatory synaptic inputs

Modulated input(s)	β Frequency	Case	Circular mean	Circular SD
SNR	12.5	Control	314.4	93.6
		6-OHDA	314.7	99.5
		+20% SNR, 6-OHDA	316.1	91.6
	25	Control	332.8	101.9
		6-OHDA	330.1	107.4
		+20% SNR, 6-OHDA	335.2	100.6
SNR + MOD + DRI-l	12.5	Control	298.8	73.4
		6-OHDA	303.3	82.5
		+20% SNR, 6-OHDA	303.3	75.8
	25	Control	306.9	74.6
		6-OHDA	308.9	85.9
		+20% SNR, 6-OHDA	311.3	78.3
SNR + MOD	12.5	Control	313.8	88.4
		6-OHDA	314.6	95.1
		+20% SNR, 6-OHDA	316.4	86.8
	25	Control	330.4	93.7
		6-OHDA	329.2	100.8
		+20% SNR, 6-OHDA	331.6	92.5
SNR + DRI-l	12.5	Control	297.4	70.8
		6-OHDA	300	80.3
		+20% SNR, 6-OHDA	300.9	72.5
	25	Control	307.6	74
		6-OHDA	307.9	85.3
		+ SNR, 6-OHDA	312.6	76.7

Next, we addressed the mechanisms underlying enhanced β oscillations in the TC neuron models. Specifically, we pursued the same approach used in a previous publication (Jaeger et al., 1997), to determine the individual contributions of membrane and postsynaptic currents during β modulation of SNR, MOD, and DRI-l inputs at 12.5 Hz (Fig. 8; same configuration shown in Fig. 7, left) and 25 Hz (Extended Data Fig. 8-1; same configuration shown in Fig. 7, right). We observed that the total postsynaptic currents induced by each group of synapses followed the β oscillations in the presynaptic activity, with the strongest component due to DRI-l inputs (Fig. 8*A*). Although no β modulation took place in the RTN synapses, the postsynaptic current displayed fluctuations, similarly to the other postsynaptic currents, resulting from variations in their driving force. Changes in driving force in turn are a result of the oscillations in the voltage membrane of the TC neuron models conveyed from the other synaptic inputs, although the total conductance of RTN synapses was kept constant over time (excluding the fluctuations due to the randomness in the presynaptic spike trains; see Fig. 3*Cb*, cyan curve). All synaptic currents were similar in normal and parkinsonian states.

To understand the role of each voltage-gated channel type in contributing to the β modulated spike rate, we plotted the β phase modulated currents averaged over all models and 10 iterations (seeds) of each model (Fig. 8*B*). We find that the outward potassium currents all show increases when the neuron is more depolarized, i.e., fires action potentials at a higher frequency. This increase is due to both an increase in driving force for potassium current and an increased activation of such voltage-gated currents induced by depolarization and spiking. Note that the increase in outward current opposes the β oscillatory membrane dynamics. In contrast, the inward calcium and sodium currents are aiding the oscillatory dynamics by their increased activation during depolarization and spiking, which leads to an increased net inward current despite a reduction in driving force for sodium and calcium ions.

To characterize the relative impact of total synaptic input currents and total voltage-gated channel currents in relaying and amplifying β oscillations, we compared the net synaptic and the net membrane currents either individually (Fig. 8*C*, blue and green curves) or summed (Fig. 8*D*, teal curves), against the instantaneous firing rates of the TC neuron models (Fig. 8*C*,*D*, black and red spike histograms in the background). By determining the total synaptic and voltage-gated current separately (Fig. 8*C*), we observed that the net currents were outward for ion channels (blue) and inward for synapses (green), respectively, throughout the entire period of β oscillation (Fig. 8*C*). The β phase locked component of the total voltage-gated current opposed the β oscillations in the synaptic input (Fig. 8*C*), overall dampening the resulting voltage oscillation. By determining the total sum of membrane and synaptic currents (Fig. 8*D*, teal), we observed that the total net current displayed more inward current as the spike rate was increasing and more outward current as the spike rate was decreasing (Fig. 8*D*, compare teal curves in the foreground against black and red spike histograms). The phases of the up and down spike rate peaks differed from the phase peaks observed in the net synaptic and the net membrane currents individually (Fig. 8*C*,*D*; compare teal curves in *D* with blue and green curves in *C*) showing that currents precede and cause membrane voltage changes (Table 6). Note that the total currents are always inward, as we are not including the leak current, which balances the mean inward current shown here. Taken together, these simulations indicate that β phase locking in voltage and spike rate are caused by the membrane response to synaptic input current, and further that the magnitude of this phase-locking is dampened by a net counterbalancing voltage-gated current, which furthermore leads to a phase shift in the voltage oscillation.

**Table 6:** Amplitudes of oscillations in membrane and synaptic currents during β modulation of synaptic inputs

β Frequency	Case	Membrane current		Synaptic current		Membrane + synaptic currents	
12.5	Control	−11.9%	+16.1%	−10.4%	+13.7%	−67.9%	+63.6%
	6-OHDA	−22.4%	+27.6%	−19.9%	+24%	−115.8%	+103.5%
25	Control	−8.6%	+11.4%	−8.6%	+8.5%	−90.4%	+101.9%
	6-OHDA	−16.1%	+19%	−14.5%	+15.9%	−151.9%	+166.6%

Numbers indicate the difference in percentage from the baseline of minimum and maximum values for down and up phases of the currents, during β modulation of modulator, nigral, and driver-like inputs.

## Discussion

We used biophysically detailed simulations to investigate the consequences of dopamine depletion on synaptic integration in VM (Fig. 1). We constructed two populations of TC neuron models reproducing the firing properties observed in VM slices from control and 6-OHDA lesioned mice (Fig. 2; Bichler et al., 2021). Thus, we have developed the first biophysically detailed model of TC neuron from VM that reproduces the differing responses observed in normal conditions and after 6-OHDA treatment. This is a substantial innovation, as the previous biophysically-detailed models of TC neurons were fitted on data of other thalamic nuclei (Iavarone et al., 2019), and they did not account for any pathologic alteration of intrinsic properties. To simulate *in vivo*-like conditions, we modeled the main afferent inputs to VM (Fig. 3), i.e., glutamatergic MOD and DRI-l, GABAergic inputs from SNR and RTN. Our simulations showed that the increased excitability observed *in vitro* after 6-OHDA lesioning (Fig. 2) also resulted in a strong firing rate increase *in vivo* (Fig. 3). The sensitivity analysis of the models of afferent inputs suggested that VM output *in vivo* is primarily driven by DRI-l and SNR inputs (Fig. 4). We then systematically tested the effects of pathologic firing pattern changes observed in dopamine-depleted conditions: synchronous bursting (Fig. 5) and increased β oscillations (Figs. 6 and 7). Synchronous SNR bursting evoked postinhibitory spike rate increases in VM TC neuron models, depending on the number of synchronous SNR inputs. The time course of firing rate increase was different in models fit to parkinsonian conditions due to reduced M-type and increased BK potassium conductances (Fig. 5). β Frequency (12.5 or 25 Hz) modulations in SNR inputs were sufficient to induce spike-phase locking in VM (Fig. 6). The resulting β modulation depth of VM spiking was amplified by the VM transfer function and was stronger than that of the SNR inputs. Adding β oscillations in MOD and DRI-l inputs enhanced this effect (Fig. 7; Table 5), primarily due to the oscillations in the DRI-l inputs (Extended Data Fig. 7-1; Table 5). Therefore, VM TC neuron models relayed and amplified the oscillatory patterns in MOD, DRI-l, and SNR inputs (Figs. 6 and 7), without differences between normal and parkinsonian states. Finally, we addressed the basic mechanisms underlying these effects, observing that they resulted primarily from the high sensitivity of the model to synaptic input current without an amplification mechanism provided by voltage-gated currents (Fig. 8; Extended Data Fig. 8-1).

### Effects of dopamine depletion *in vitro*

*In vitro* recordings showed that dopamine depletion increased excitability of VM as a result of M-type current suppression (Bichler et al., 2021). Comparing the parameters of normal and parkinsonian models, we determined differences in their T-type and M-type currents. In particular, the M-type conductance was 32-fold lower in parkinsonian models than control, and a subsequent 40-fold increase in M-type conductance yielded f-I curves and rebound spike counts in parkinsonian models indistinguishable from control. M-type current in thalamus is normally regulated by acetylcholine, where the activation of muscarinic receptors depolarizes thalamic neurons during high-attentional states (Llinás and Steriade, 2006). The main source of VM cholinergic input is from the pedunculopontine tegmentum and the laterodorsal tegmental nucleus (Huerta-Ocampo et al., 2020), and optogenetic stimulation of cholinergic neurons in this area switches sleep states from NREM to REM in mice (Van Dort et al., 2015). Indeed, optogenetic activation of VM neurons directly promotes arousal and wakes mice from NREM sleep (Honjoh et al., 2018). Therefore, we speculate that M-type current downregulation in VM would be causative of disturbed sleep states in PD that are characterized by insomnia, increased arousal and restless leg syndrome (for review, see Lajoie et al., 2021). In agreement with our simulations, muscarinic positive allosteric modulators that enhance M-type (Kv7) currents might have anti-parkinsonian effects on motor thalamic activity in PD and might improve associated sleep disorders.

### Effects of dopamine depletion on thalamic firing rates *in vivo*

Our results indicated that firing rates in response to simulated *in vivo*-like inputs in the parkinsonian VM TC neuron models were robustly increased compared with control models due to M-type potassium current reduction. However, robust rate increases are not observed in thalamic recordings from dopamine-depleted rodents *in vivo* (Anderson et al., 2015; Di Giovanni et al., 2020; Nakamura et al., 2021). As classic models suggested that motor deficits in PD might be due to increased basal ganglia inhibition to motor thalamus, and that this might cause motor deficits (DeLong, 1990), we tested the hypothesis that increased inhibition in conjunction with increased excitability could lead to a normalization in firing rates. Indeed, increasing the conductance of SNR synapses by 20% yielded firing rates in parkinsonian models indistinguishable from control. Therefore, increased excitability of VM neurons after dopamine depletion might be a compensatory mechanism for increased inhibition in this state.

Our sensitivity analysis of the effect of different input sources on VM firing suggested that VM activity *in vivo* was primarily influenced by SNR inhibition, whereas the impact of RTN inhibition was weak (Fig. 4). This was primarily due to the smaller size and more distal location of RTN terminals that we adopted from the findings of anatomic studies (Ilinsky and Kultas‐Ilinsky, 2002; Wanaverbecq et al., 2008). This is consistent with experimental findings showing that the impact of non-nigral inhibition on VM of mouse was not substantial (Chevalier and Deniau, 1982).

### Impact of nigral bursting on motor thalamic activity

*In vitro* studies demonstrated that synchronous SNR activation could evoke rebound bursting based on T-type Ca^2+^ current activation in VM (Edgerton and Jaeger, 2014). However, these experiments were conducted in the absence of background *in vivo* excitatory inputs that will limit the hyperpolarization achieved by nigral bursts. This effect of excitatory inputs was borne out by our simulations, where even 100% of synchronous SNR bursting in the presence of a tonic background of inhibition and excitation did not allow the development of T-type rebound bursts (Fig. 5). Instead, we found that under these conditions M-type and BK potassium currents caused a slower postinhibitory firing increase in VM neurons that was correlated with the percentage of synchronous SNR inputs (Fig. 5). Moreover, in our simulations, identical SNR inputs evoked rebound spiking with different courses in normal and parkinsonian states (Fig. 5), due to changes in M-type and BK current activation. This suggests that an altered course of postinhibitory spiking could be a marker of parkinsonian pathophysiology in VM as a consequence to excitability changes induced by dopamine depletion.

### Impact of β oscillations in the afferent inputs to VM

*In vivo* recordings showed that dopamine depletion enhanced β oscillations in motor areas and that VM promoted their propagation to MC (Brazhnik et al., 2012, 2016). We simulated β oscillations at 12.5 or 25 Hz as a spike-phase locking in the activity of SNR input to VM, and/or cortical MOD and DRI-l inputs, consistent with the experimental recordings (Brazhnik et al., 2012, 2016). Particularly, recordings showed that β band oscillations were prevalent in nonattentive resting states whereas increased β oscillations occurred during treadmill walking (Brazhnik et al., 2012). In our simulations, low-amplitude β oscillations in the afferent inputs resulted in significant spike-phase locking in VM at both 12.5 and 25 Hz (Figs. 6 and 7), which not only relayed but also amplified the oscillatory patterns in input (Figs. 6 and 7). This amplification was not different between normal and parkinsonian conditions. Therefore, our simulation results suggest that the increased β oscillations and spike-phase locking observed in dopamine-depleted animals (Brazhnik et al., 2016; Nakamura et al., 2021) is a consequence of changes in the activity of the afferent inputs to VM and do not rely on intrinsic excitability changes in VM.

*In vivo* experiments showed the necessity of SNR-VM interactions for propagation of β oscillations to MC (Brazhnik et al., 2016). Our simulations showed that β oscillations in SNR alone were sufficient to induce significant spike-phase locking in VM (Fig. 6), supporting the possibility that β oscillations could originate in SNR and then entrain the cortico-thalamocortical loop through the VM. However, results in dopamine-depleted mice indicate a lack of β frequency increases in SNR (Lobb and Jaeger, 2015; Willard et al., 2019), suggesting a species-specific or state-dependent outcome in this regard.

In conclusion, modeling motor thalamic synaptic integration in normal and parkinsonian states indicates that parkinsonian firing pattern changes in the inputs are readily transmitted to the outputs. Increased intrinsic excitability may primarily act to compensate for changes in the excitatory/inhibitory input balance and lead to a homeostatic recalibration of firing rates. The increased excitability may come at the cost of accurate cholinergic modulation in motor thalamus, however, and contribute fluctuations in cortical arousal.

## Synthesis

Reviewing Editor: Arvind Kumar, KTH Royal Institute of Technology Decisions are customarily a result of the Reviewing Editor and the peer reviewers coming together and discussing their recommendations until a consensus is reached. When revisions are invited, a fact-based synthesis statement explaining their decision and outlining what is needed to prepare a revision will be listed below. The following reviewer(s) agreed to reveal their identity: Hajnalka Bokor, Robert Schmidt. Note: If this manuscript was transferred from JNeurosci and a decision was made to accept the manuscript without peer review, a brief statement to this effect will instead be what is listed below.

Synthesis

In this manuscript the authors report the behavior of a biophysical model of a thalamic neuron. Authors have done an impressive job in integrating different data to build the model and characterize the model for propagation of beta oscillations.

The manuscript was reviewed by two reviewers. We were lucky to have one reviewer from the previous round at J. Neurosci. While the first reviewer is happy with the revision, the second reviewer has raised several concerns which we think can be addressed in a revision. In particular we would like to address the following key issues.

Provide further details of the model e.g diff equations, how the parameters were selected and in what ways normal and PD models differ. The difference in parameter values can have consequences for sensitivity analysis [see comment 5 by the second reviewer]

Given the number of free parameters the match between simulation and experimental data is not very good. One can say that there is even a qualitative difference e,g the f-i curves Fig 2B. This should be discussed if not systematically investigated.

Some of the conclusions may need further discussion and in their current form thay may not be entirely true - e.g ‘confirms that SNR and DRIs are the major contributor to the spike‐phase locking’ [see comment 5 by the second reviewer]

Discuss the mechanism that underlies the enhancement of beta oscillations [see comment 8 by the second reviewer] - this could form the basis of testable predictions

The detailed comments of the reviewers are appended below to help you prepare the revision and your rebuttal.

Reviewer #1

The authors considered and tackled all points raised in the review and their answers are appropriate and clear, below I have indicated a minor further comment concerning their responses. The paper improved significantly with the additional figures and clarifications made in the text. I have no further major questions or comments for the authors.

minor:

1) EM method (from line 412) The description is still somewhat confusing. State clearly that singe section EM sections were used to estimate synaptic coverage of VM model cells. Line 422-423: how can active zones be that long for mod and RTN terminals? Did you mean bouton size, if yes how was that measured? In case of the nigral terminals the data from Bodor et al. 2.8 μm is the average bouton size, not the average diameter of the active zone.

2) gabaergic, change to GABAergic

Reviewer #2

In their manuscript “Modeling synaptic integration of bursty and beta oscillatory inputs in ventromedial motor thalamic neurons in normal and parkinsonian states” the authors develop a detailed model of a TC neuron and study its behaviour under Normal and Parkinsonian conditions in relation to in-vitro stimulation protocols and in-vivo synaptic inputs. The models are the result of an elaborate fitting procedure utilizing existing experimental data. For the simulations of in-vivo conditions the activity of the model neurons is examined for bursting activity in SNR as well as beta-modulated inputs from SNR and other regions. Main results include that TC model neurons enhance beta oscillations present in their inputs; dopamine-depletion leads to increased activity in TC neurons; and TC post-inhibitory firing rate increases are due to M-type potassium channels and are prolonged in Parkinsonian conditions.

Strengths of the paper include the impressive effort to incorporate a large amount of experimental data to generate and test detailed models of TC neurons. Furthermore, the model is used to examine important questions about beta oscillation propagation and activity changes related to Parkinson’s disease.

I see several major issues that relate to the model and the interpretation of the results:

1. I think more information about the model(s) needs to be provided. There is little information on the parameters of the models that were selected. It would be good to summarize the model equations e.g. in a table. I would appreciate if the model equations are provided instead of pointing to other papers. For example, I tried to look up the equations for the HH conductances in Iavarone et al. (2019). In there for some of the conductances again other papers are cited. It gets really difficult to see what was actually used. Currently, it is also difficult for the reader to identify the free parameters in the current equations mentioned in the “Fitting neuron models” section. The Parkinson models had different parameters than the Healthy models, so it would be good to also see those and compare how they differed (lines 788-793: Where can the mentioned comparison of the normal and parkinsonian model parameters be found in the paper?). I don’t think it is sufficient to just point the reader to the GitHub implementation to look up all this information themselves, it should be provided in the main paper somewhere (the GitHub link is also not working).

2. It seems that for both Normal and Parkinsonian conditions a set of models passed all the applied criteria. How many models were there in each case and what were their parameter values or ranges? As far as I understand it, the authors report some results for the model population (e.g. the distributions shown in Fig. 2-2, or in Fig. 4), but for other simulation results one particular model instance is selected (Figs. 5-7). Please clarify this.

3. Is the set of models meant to reflect the variability present across TC neurons? If so, then the distributions shown in Fig. 2-2 look quite different comparing experiment and model data. Please clarify what the different data points are in Fig. 2-2, are these different stimulation protocols, or different model instances?

4. Fig. 2A (and extended figures): Why are different input currents shown for experiment and simulation data? I thought the point here was to demonstrate the model(s) reproduce the experimental traces very well? I also agree with the previous reviewer that the simulations seem to look different from the experiments. The author’s response emphasized the variability present in the population. However, the shown traces rather emphasize that some properties are not captured well by the model, the distributions in Fig. 2-2 also look quite different (sometimes similar median, but overall very different shapes), and in Fig. 2D the model spike count for the control with 20-40mV is quite different from the data). I guess overall my impression was that for a detailed neuron model with some many free parameters, the fit to the experimental data seems a bit underwhelming.

5. Related to a point previously brought up by another reviewer: the fitting procedure yielded neuron in-vivo TC models with a baseline firing rate of ∼200 Hz (l. 441). To correct this, the authors increased the number of SNR inputs from 25 to 100. This seems to be a major change that is likely to have affected the subsequent results, e.g. on the effect of beta propagation from SNR. An alternative configuration is mentioned (l. 447), but I did not find a reference to the new Fig 7-3 in the text. I did not understand the author’s response that Fig 7-3 “confirms that SNR and DRIs are the major contributor to the spike‐phase locking”. Couldn’t it be just DRI causing the phase-locking in that case? Furthermore, also the previous results might be affected by this change (Figs 3-5). It is mentioned that “the highest values of total synaptic conductance over time were achieved by SNR inputs and DRI-l (Fig. 3Cb), suggesting that these two classes of synaptic inputs sustained the TC neuron firing in normal and parkinsonian states”, which seems then to be a direct consequence of the increased number of SNR inputs, rather than a finding. Or would this not change in the alternative configuration? In addition, I think the adjustment to the number of SNR neurons need to be mentioned also in the Results and/or Discussion as this seems to be a key point where the model deviates from currently available experimental data.

A related point: in the sensitivity analyses (Fig. 4) the considered variation is done within +-10%. So if I understand it correctly, the number of SNR inputs would be varied from 90 to 110. Given the large adjustment that needed to be done for the number of SNR inputs (ie from 25 to 100), this seems to be a rather small range. So my conclusion would be that this does not exclude that there are other regions of the parameter space where the other inputs (RTN, MOD) would have a larger effect. Anyway, I think my point is that the section claims “To establish the importance of each synaptic afferent”, but to me it seems that the importance of the SNR afferents had already been build-in to the model above.

6. The reversal potential for inhibitory synapses (so also for SNR-TC) was set to -76.4 mV (line 399). The given reference (Pathak et al., 2007) seems to apply to hippocampal granule cells. There is evidence that the reversal potential is actually more negative and this has been taken as part of the proposed mechanism for how T-type Ca channels can support postinhibitory rebound spiking (e.g. Huguenard, J. R., & Prince, D. A. (1994). Intrathalamic rhythmicity studied in vitro: nominal T-current modulation causes robust antioscillatory effects. Journal of Neuroscience, 14(9), 5485-5502. Ulrich, D., & Huguenard, J. R. (1997). Nucleus-specific chloride homeostasis in rat thalamus. Journal of Neuroscience, 17(7), 2348-2354.). Therefore it seems the finding that the T-type Ca channels do not seem to be relevant for the postinhibitory firing rate increase (Fig. 5) is a direct consequence of the reversal potential parameter setting.

Therefore, I am also not sure about the explanation (line 832) that the lack of excitatory inputs explains the absence of T-type rebound bursts.

7. In Figure 5D the Control model produces a higher post-inhibitory firing rate increase (∼+65Hz) than the parkinson model (∼+40Hz). The authors report that the time courses are different (longer in the parkinson model), but the apparently quite strong differences in the amplitude of the response are not mentioned. This finding seems at odds with rebound spiking being more prominent in parkinson states.

8. One of the main results is that TC model neurons seem to enhance beta oscillations. What is the underlying mechanism of that effect in the detailed neuron model?

Minor issues:

- In Table 2 protocol 2 is missing. For some the variable names in the source code are provided, for others they are not. For some there are explanations about their meaning or how they are calculated, for others there are none. Please try to include the relevant information here consistently.

- Please clarify how your model relates to similar previous models (Iavarone et al., 2019). What are the differences and what was the reasoning to develop another detailed model, rather than using an existing model?

- line 360: it seems quite generous to allow errors to fall within 3 standard deviations? Related to Major Point 1, what % of models made it to the Hall of Fame? How many model candidates passed all the subsequent quality checks?

- line 328: typo, probably should read: “... dynamics of NaT, KDR, KM, and BK currents, ...”?

- line 729: I don’t think the colors mentioned here match those of the Figure

- line 757: I think this should refer to Table 4 instead of Table 3

- line 741: “enhanced the significance of the spike-phase coherence (Rayleigh, p < 0.001)” This is phrased as if p-values were statistically compared (“enhanced”), but it seems that the test confirmed that the spike phase distribution is not uniform? Also it might be more accurate to refer to phase-locking here rather than spike-phase coherence.

## Author Response

In this manuscript the authors report the behavior of a biophysical model of a thalamic neuron. Authors have done an impressive job in integrating different data to build the model and characterize the model for propagation of beta oscillations.

The manuscript was reviewed by two reviewers. We were lucky to have one reviewer from the previous round at J. Neurosci. While the first reviewer is happy with the revision, the second reviewer has raised several concerns which we think can be addressed in a revision. In particular we would like to address the following key issues.

Provide further details of the model e.g diff equations, how the parameters were selected and in what ways normal and PD models differ. The difference in parameter values can have consequences for sensitivity analysis [see comment 5 by the second reviewer]

Given the number of free parameters the match between simulation and experimental data is not very good. One can say that there is even a qualitative difference e,g the f-i curves Fig 2B. This should be discussed if not systematically investigated.

Some of the conclusions may need further discussion and in their current form thay may not be entirely true - e.g ‘confirms that SNR and DRIs are the major contributor to the spike‐phase locking’ [see comment 5 by the second reviewer]

Discuss the mechanism that underlies the enhancement of beta oscillations [see comment 8 by the second reviewer] - this could form the basis of testable predictions

The detailed comments of the reviewers are appended below to help you prepare the revision and your rebuttal.

Summary: We would like to thank the editor and both the reviewers for their suggestions as well as comments. By addressing their questions, the results reported in our manuscript have been strengthened. Please, see below for reviewer comments (in blue) and our detailed responses (in black). We made substantial edits to address the comments of both reviewers, while we ran additional simulations and performed additional analyses of the data to address the concerns expressed by the second reviewer. Additionally, we have revised Fig. 2-2, replacing the AP accommodation (Fig. 2-2, bottom, left panel) with the firing rate adaptation, measured experimentally and using simulations from TC neurons in normal and parkinsonian states. This change makes the manuscript more consistent, as we preferred to show the same firing properties used as targets during model fitting. We have also corrected the number of free parameters (line 324 in the previous version of the manuscript) and the numbers of optimization sessions ran to fit the TC neuron models in normal conditions (lines 350-351 in the previous version of the manuscript) reported in the previous version of the manuscript.

Reviewer #1

The authors considered and tackled all points raised in the review and their answers are appropriate and clear, below I have indicated a minor further comment concerning their responses. The paper improved significantly with the additional figures and clarifications made in the text. I have no further major questions or comments for the authors.

We thank the reviewer for their suggestions and acknowledging the improvements of our manuscript.

minor:

1) EM method (from line 412) The description is still somewhat confusing. State clearly that singe section EM sections were used to estimate synaptic coverage of VM model cells. Line 422-423: how can active zones be that long for mod and RTN terminals? Did you mean bouton size, if yes how was that measured? In case of the nigral terminals the data from Bodor et al. 2.8 μm is the average bouton size, not the average diameter of the active zone.

We now state clearly that single EM sections were used for estimating the synaptic coverage of VM model cells. Additionally, we confirm that 0.8 μm was the average size of terminals for corticothalamic and reticular inputs, while 2.8 μm was the average size of the longest diameter of nigrothalamic terminals. Therefore, we have updated the manuscript accordingly.

2) gabaergic, change to GABAergic

Thanks. Done.

Reviewer #2

In their manuscript “Modeling synaptic integration of bursty and beta oscillatory inputs in ventromedial motor thalamic neurons in normal and parkinsonian states” the authors develop a detailed model of a TC neuron and study its behaviour under Normal and Parkinsonian conditions in relation to in-vitro stimulation protocols and in-vivo synaptic inputs. The models are the result of an elaborate fitting procedure utilizing existing experimental data. For the simulations of in-vivo conditions the activity of the model neurons is examined for bursting activity in SNR as well as beta-modulated inputs from SNR and other regions. Main results include that TC model neurons enhance beta oscillations present in their inputs; dopamine-depletion leads to increased activity in TC neurons; and TC post-inhibitory firing rate increases are due to M-type potassium channels and are prolonged in Parkinsonian conditions.

Strengths of the paper include the impressive effort to incorporate a large amount of experimental data to generate and test detailed models of TC neurons. Furthermore, the model is used to examine important questions about beta oscillation propagation and activity changes related to Parkinson’s disease.

We agree with the summary provided by the reviewer and thank them for acknowledging our efforts in incorporating a large amount of experimental data into our model of thalamocortical neuron. To address the concerns of the second reviewer, we have updated the configuration of synaptic inputs to VM as follows:

1. We have reduced by ∼25% the synaptic conductance peak of the driver-like inputs to approximate the short-term depression, i.e., paired-pulse ratio, observed in VM slices of mice (Gornati et al, 2018), pursuing the same modeling approach shown in a previous publication (Abbasi et al, 2017).

2. We decreased the GABA reversal potential to -81 mV, the value observed in other areas of thalamus (see point 6).

3. We used 25 SNR inputs instead of 100 (see point 5), according to our initial estimates from experimental data. However, we have increased the synaptic conductance peak for SNR inputs by 4.25 times, now using a value of 4.8 nS, which is compatible with previous experimental estimates (Edgerton and Jaeger, 2014).

I see several major issues that relate to the model and the interpretation of the results:

1. I think more information about the model(s) needs to be provided. There is little information on the parameters of the models that were selected. It would be good to summarize the model equations e.g. in a table. I would appreciate if the model equations are provided instead of pointing to other papers. For example, I tried to look up the equations for the HH conductances in Iavarone et al. (2019). In there for some of the conductances again other papers are cited. It gets really difficult to see what was actually used. Currently, it is also difficult for the reader to identify the free parameters in the current equations mentioned in the “Fitting neuron models” section. The Parkinson models had different parameters than the Healthy models, so it would be good to also see those and compare how they differed (lines 788-793: Where can the mentioned comparison of the normal and parkinsonian model parameters be found in the paper?). I don’t think it is sufficient to just point the reader to the GitHub implementation to look up all this information themselves, it should be provided in the main paper somewhere (the GitHub link is also not working).

We have added an extended table (Table 3-1) to show the HH equations describing the membrane properties of our neuron models. In this table, the terms treated as free parameters are marked in bold. Additionally, the free parameters are listed in Table 3. Moreover, we note that, for both normal and parkinsonian models, we have defined the same sets of free parameters, and the optimizer searched throughout the same parameter ranges. However, as an emergent property, the distributions are significantly different between normal and parkinsonian conditions for three parameters related to T-type calcium and M-type potassium currents (see Table 3, asterisks). To clarify this, we have created a table describing mean +/- mean standard error (SE) of the free parameters in both states, marking those that are statistically different in normal and parkinsonian states (see Table 3, asterisks). Finally, we have set the access of the Github repository to ‘public’ so that the link is now working.

2. It seems that for both Normal and Parkinsonian conditions a set of models passed all the applied criteria. How many models were there in each case and what were their parameter values or ranges? As far as I understand it, the authors report some results for the model population (e.g. the distributions shown in Fig. 2-2, or in Fig. 4), but for other simulation results one particular model instance is selected (Figs. 5-7). Please clarify this.

In Materials and Methods, we reported (between parentheses) the numbers of normal and parkinsonian models that passed each quality check (see lines 360-378 and 360-380 in the previous and current versions of the manuscript, respectively). We have also added a Table showing the distributions of the parameters for the models that have passed all the quality checks (see Table 3), and then used to simulate in vivo-like conditions (Figs. 3-7).

In Figures 5-7 (of the previous version of the manuscript), including the related Supplementary Figures, we showed the pooled responses of the models generated with 10 simulations per model, where synaptic inputs were randomly generated with different seeds. These pooled responses were shown in the spike time histograms along with the corresponding raster plots in Figs 5-7. Similarly, the curves of the transmembrane currents shown in Fig. 5D represent the average across all the models generating a significant post-inhibitory firing rate increase and 10 simulations per model. Instead, Fig. 5A showed the responses for particular models.

We have revised Figs 5-7 and the related Supplementary Figures. In the new version of these figures, the spike time histograms are calculated from the pooled responses of TC neuron models, obtained with 10 simulations per model (same as before). Additionally, the raster plots show representative responses generated by subsets of TC neuron models in normal and parkinsonian states, obtained with a single simulation per model. This change in the raster plots has improved the visibility of the firing activity generated by different TC neuron models. Therefore, the spike time histograms provide general information about the entire model population as well as synaptic inputs, while the raster plots facilitate the visual recognition of the firing patterns exhibited in the model responses. Moreover, Fig. 5A shows the responses for particular models, the same way as before, to give an impression of the post-inhibitory firing rate following synchronous SNR bursting. Finally, we have revised the figure captions to match the new version of Figs. 5-7, and to address the initial concern of the reviewer, clarifying how many models and simulations per model are shown in each spike histogram and raster plot.

3. Is the set of models meant to reflect the variability present across TC neurons? If so, then the distributions shown in Fig. 2-2 look quite different comparing experiment and model data. Please clarify what the different data points are in Fig. 2-2, are these different stimulation protocols, or different model instances?

We did not aim to generate a population of models that reproduced the exact experimental distributions of the firing properties. The optimizer rather yielded model populations that minimized the mean squared error associated with the firing properties observed experimentally. Therefore, we fitted neuron models that displayed a behavior within the physiological ranges, as exemplified by the firing properties shown in Fig. 2-2, even though the population of models does not replicate the exact distributions of properties observed experimentally. The replication of the experimental distributions of the firing properties is beyond the scope of our project, and to our knowledge has never been aimed at with similar optimization techniques. However, as intended, our model selection yielded a subset of models displaying all optimized measures within three standard deviations from the mean (i.e., mean and standard deviation estimated experimentally).

With regard to Fig. 2-2, the data points in the violin plots were not individually visible, and further the violin plots showed a distribution even below the lowest data value due to how this Python function estimates distributions from values. We now show the data as box plots that represent the values of the firing properties, and each point corresponds to the values observed in a single cell (blue) and/or single model (orange). On top of the boxplot, we also show the data points (black) used to generate the boxplots. To measure the firing properties, we used somatic current injections in the experiments, while the simulation faithfully reproduces the same experimental protocols and conditions. The protocols used or simulated are described in section “Template data taken from in vitro experiments”. We have revised the figure caption to make this point clear.

4. Fig. 2A (and extended figures): Why are different input currents shown for experiment and simulation data? I thought the point here was to demonstrate the model(s) reproduce the experimental traces very well? I also agree with the previous reviewer that the simulations seem to look different from the experiments. The author’s response emphasized the variability present in the population. However, the shown traces rather emphasize that some properties are not captured well by the model, the distributions in Fig. 2-2 also look quite different (sometimes similar median, but overall very different shapes), and in Fig. 2D the model spike count for the control with 20-40mV is quite different from the data). I guess overall my impression was that for a detailed neuron model with some many free parameters, the fit to the experimental data seems a bit underwhelming.

Figure 2A shows a single representative experimental and simulation trace that displays similar firing behaviors at similar current steps. Although the step currents applied in experiments and simulations were not the same, they differ by only 20 pA. Our optimization approach was aimed at fitting within the three standard deviations of the experimental variability, and thus the models themselves do not match the response of any neuron. Moreover, we agree that the shape of the distributions shown in Fig. 2-2 (ref. violin and box plots in previous and current version of the manuscript, respectively) look quite different for experiments and simulations. As mentioned above, the optimizer did not aim to match the distribution shapes of the firing properties considered as targets. Instead, as intended, the medians were statistically indistinguishable for most of the action potential and membrane properties shown in Fig. 2-2, except the action potential amplitude, which was lower in simulations than experiments. Therefore, we agree with the reviewer that more could be done to obtain better models of ventromedial thalamocortical neurons. For example, we tended to not optimize the dynamics of the ion channels, and only a minority of their parameters (i.e., variables in the ion channel equations) were treated as free parameters. We believe that optimizing the ion channel dynamics more fully could greatly improve the realism and accuracy of TC neuron models. In fact, one of the previous modeling papers from the Jaeger lab (Hendrickson, E., J. Edgerton and D. Jaeger (2011). “The use of automated parameter searches to improve ion channel kinetics for neural modeling.” Journal of Computational Neuroscience: 1-18) showed exactly that a 2-stage optimization design that sets kinetic variables free in the 2nd stage outperforms the commonly used strategy of only adjusting channel densities. Therefore, the current version of our models could likely be improved for parameters like detailed action potential and AHP shapes, though in our experience that has little influence on synaptic integration of in vivo-like input patterns.

5. Related to a point previously brought up by another reviewer: the fitting procedure yielded neuron in-vivo TC models with a baseline firing rate of ∼200 Hz (l. 441). To correct this, the authors increased the number of SNR inputs from 25 to 100. This seems to be a major change that is likely to have affected the subsequent results, e.g. on the effect of beta propagation from SNR. An alternative configuration is mentioned (l. 447), but I did not find a reference to the new Fig 7-3 in the text. I did not understand the author’s response that Fig 7-3 “confirms that SNR and DRIs are the major contributor to the spike‐phase locking”. Couldn’t it be just DRI causing the phase-locking in that case? Furthermore, also the previous results might be affected by this change (Figs 3-5). It is mentioned that “the highest values of total synaptic conductance over time were achieved by SNR inputs and DRI-l (Fig. 3Cb), suggesting that these two classes of synaptic inputs sustained the TC neuron firing in normal and parkinsonian states”, which seems then to be a direct consequence of the increased number of SNR inputs, rather than a finding. Or would this not change in the alternative configuration? In addition, I think the adjustment to the number of SNR neurons need to be mentioned also in the Results and/or Discussion as this seems to be a key point where the model deviates from currently available experimental data.

A related point: in the sensitivity analyses (Fig. 4) the considered variation is done within +-10%. So if I understand it correctly, the number of SNR inputs would be varied from 90 to 110. Given the large adjustment that needed to be done for the number of SNR inputs (ie from 25 to 100), this seems to be a rather small range. So my conclusion would be that this does not exclude that there are other regions of the parameter space where the other inputs (RTN, MOD) would have a larger effect. Anyway, I think my point is that the section claims “To establish the importance of each synaptic afferent”, but to me it seems that the importance of the SNR afferents had already been build-in to the model above.

We have taken into account the concerns expressed by the reviewer regarding the increased number of SNR inputs from 25 to 100. Therefore, we updated the model, using a new configuration with 25 SNR inputs throughout. Additionally, we now use a GABA reversal potential of -81 mV (see point 6). Compared to the previous configuration, the SNR conductance was increased by 4.25 times, resulting in a value that is still in agreement with the range of experimental data (Edgerton and Jaeger, 2014). In fact, the new unitary conductance of 4.8 nS is clearly within the physiological range of strong GABA synapses. Using this new configuration of synaptic inputs, we have performed the same simulations as before (updated results shown in new Figs. 3 to 7), confirming the results given in the previous version of the manuscript with only minor deviations. We repeated the sensitivity analysis (Fig. 3), confirming that the conclusion that DRI-l and SNR input are the most influential inputs to VM still holds with 25 SNR inputs. Taken together, these results do not support the initial hypothesis of the reviewer, that the importance of the SNR afferents was a “build-in” feature due to the 4-folds increase in the number of SNR synapses. As we now used 25 SNR inputs for our standard synaptic configuration, we deleted Figure 7-3, which was our previous ‘alternative configuration’ with 25 SNR inputs instead of 100.

We agree with the reviewer that there may be other regions of the parameter space in which RTN and MOD might have a stronger impact on the TC firing. Specifically, these would be regions where these inputs have a much higher total conductance, which however we could not find any experimental evidence for.

6. The reversal potential for inhibitory synapses (so also for SNR-TC) was set to -76.4 mV (line 399). The given reference (Pathak et al., 2007) seems to apply to hippocampal granule cells. There is evidence that the reversal potential is actually more negative and this has been taken as part of the proposed mechanism for how T-type Ca channels can support postinhibitory rebound spiking (e.g. Huguenard, J. R., & Prince, D. A. (1994). Intrathalamic rhythmicity studied in vitro: nominal T-current modulation causes robust antioscillatory effects. Journal of Neuroscience, 14(9), 5485-5502. Ulrich, D., & Huguenard, J. R. (1997). Nucleus-specific chloride homeostasis in rat thalamus. Journal of Neuroscience, 17(7), 2348-2354.). Therefore it seems the finding that the T-type Ca channels do not seem to be relevant for the postinhibitory firing rate increase (Fig. 5) is a direct consequence of the reversal potential parameter setting. Therefore, I am also not sure about the explanation (line 832) that the lack of excitatory inputs explains the absence of T-type rebound bursts.

We have followed the suggestion provided by the reviewer, using a value of the GABA reversal potential recorded in thalamus (-81 mV; see Ulrich & Huguenard, 1997). As stated above, we also now set the number of SNR synapses to 25, and estimated the conductance value that was required to yield realistic spike rates in conjunction with DRI and MOD excitation. This still yielded physiological values of unitary conductance amplitudes (see point 5). With this updated configuration, synchronous nigral bursting induced a hyperpolarization peak of -75 mV (see Fig. 5A), lower than observed with the previous configuration of SNR inputs (-70 mV). However, this hyperpolarization is still insufficient to reset the T-type Ca2+ channel, and synchronous nigral bursting was still unable to evoke rebound bursting in the TC neuron models (see Fig. 5D). We note that we have tested the impact of a configuration with a reversal potential of -81 mV for GABA and 100 SNR synapses, obtaining the same results (not shown). Therefore, our model suggests that the lack of rebound bursting in response to synchronous nigral inputs was not an artificial consequence due to the relatively depolarized value of GABA reversal potential. It should be noted, however, that homeostatic regulation of internal chloride concentration in a thalamic structure receiving an additional major source of GABA inhibition compared to sensory thalamic nuclei might in fact result in a more depolarized E-GABA. This has never been carefully measured in VM thalamus, however.

7. In Figure 5D the Control model produces a higher post-inhibitory firing rate increase (∼+65Hz) than the parkinson model (∼+40Hz). The authors report that the time courses are different (longer in the parkinson model), but the apparently quite strong differences in the amplitude of the response are not mentioned. This finding seems at odds with rebound spiking being more prominent in parkinson states.

In this part of the manuscript, we have shown the impact of synchronous nigral bursting on TC neuron activity in “in vivo-like” conditions. In addressing this question, we did not make any assumption, nor aimed to prove that rebound bursting is stronger in parkinsonian state than control in vivo. Therefore, the model suggests that TC neurons display different response to synchronous nigral bursting, due to the activation of different membrane mechanisms, i.e., M-type and BK potassium, respectively. In the new version of the model, with updated reversal potential, synaptic conductances for the GABAergic inputs, and number of SNR synapses, these findings remain confirmed. These findings suggest that increased thalamic bursting in parkinsonian states is a result of changed input patterns rather than of changed intrinsic properties.

8. One of the main results is that TC model neurons seem to enhance beta oscillations. What is the underlying mechanism of that effect in the detailed neuron model?

To address this question, we now analyzed the variations of membrane and postsynaptic currents against the instantaneous firing rate of the TC neuron models during a beta oscillation cycle, following the same approach shown in a previous modeling paper (Jaeger et al, 1997). This led to a new main figure in the manuscript, figure 8, and a new Supplementary figure, figure 8-1. We have found that the amplification of beta oscillations resulted directly from synaptic currents, with voltage-gated currents actually opposing the synaptic influence. Additionally, we have found that the combined effects of ion channels influenced the phase of the beta oscillations in the firing rate of the TC neuron models. We thank the reviewer for encouraging this important additional analysis.

Minor issues:

- In Table 2 protocol 2 is missing. For some the variable names in the source code are provided, for others they are not. For some there are explanations about their meaning or how they are calculated, for others there are none. Please try to include the relevant information here consistently.

Table 2 describes the firing features that constitute the targets of our model optimization, along with the protocols used for their estimation in both experiments and simulations. As Protocol 2 was not used to estimate any target used in the optimization (but rather to perform the quality checks; see lines 361-366 and lines 361-362 in the previous and current version of the manuscript, respectively), it is not listed in Table 2.

We chose to limit the explanations to those features whose meaning or calculation were not common knowledge, nor inferable from their names. To not overcrowd the table, we omitted the calculation of self-explanatory parameters such as AP amplitude and AP count. Note that the exact calculations are best seen by consulting the source code (now available as public) and are largely derived from a published library (related to BluPyOpt: the eFEL python package aka Electrophys Feature Extraction Library; see https://efel.readthedocs.io/en/latest/eFeatures.html; for a fully detailed description of firing property measures, see also http://bluebrain.github.io/eFEL/efeature-documentation.pdf)

- Please clarify how your model relates to similar previous models (Iavarone et al., 2019). What are the differences and what was the reasoning to develop another detailed model, rather than using an existing model?

Our thalamocortical neuron model is fitted on data of ventromedial motor thalamus in normal conditions and after dopamine depletion. To the best of our knowledge, our model is the first one replicating the responses observed in both normal and parkinsonian conditions. Additionally, the existing thalamocortical neuron models (e.g., Iavarone et al., 2019) were fitted on data of ventrobasal thalamus. We have clarified this point in the discussions.

- line 360: it seems quite generous to allow errors to fall within 3 standard deviations? Related to Major Point 1, what % of models made it to the Hall of Fame? How many model candidates passed all the subsequent quality checks?

The Hall-of-Fame are models whose firing properties fell within the three standard deviations threshold (for details, see Materials and Methods). This can be seen as the first quality check. The other models, which do not pass this quality check, can be considered as generating non-physiological responses, and thus we prefer to not include them in further analyses. Therefore, we have indicated the numbers of hall-of-fame without considering their percent with respect to the totality of the models tested by the optimizer (which is quite large; Control: n=450000 and 6-OHDA: n=150000 models), followed by the numbers of models which passed each of the other quality checks, as indicated in lines 359-378 (in the previous version of the manuscript; lines 360-380 in the current version of the manuscripts).

- line 328: typo, probably should read: “... dynamics of NaT, KDR, KM, and BK currents, ...”?

Thanks. Fixed.

- line 729: I don’t think the colors mentioned here match those of the Figure

Thanks. Fixed.

- line 757: I think this should refer to Table 4 instead of Table 3

By adding Table 3 (see points 1 & 2), the numbers were increased for all the Tables. Therefore, Table 4 in the previous version of the manuscript corresponds to Table 5 in the current version of the manuscript. All table references should now be updated and accurate.

- line 741: “enhanced the significance of the spike-phase coherence (Rayleigh, p < 0.001)” This is phrased as if p-values were statistically compared (“enhanced”), but it seems that the test confirmed that the spike phase distribution is not uniform? Also it might be more accurate to refer to phase-locking here rather than spike-phase coherence.

We tested the spike-phase locking by introducing beta-modulation in each synaptic group individually, observing that each synaptic input yielded significant spike-phase locking (see Fig. 6-1). We agree that comparison between p-values obtained with different oscillatory inputs is not the best approach to evaluate the spike-phase locking induced by each. We prefer to compare the circular variance obtained with each beta modulated input. We have also revised the text to make this point clear. We have also replaced “spike-phase coherence” with “spike-phase locking” for consistency.
