# Supplementary figures and images for: Modeling Synaptic Integration of Bursty and β Oscillatory Inputs in Ventromedial Motor Thalamic Neurons in Normal and Parkinsonian States
Source: eNeuro. 2023 Dec 8;10(12):ENEURO.0237-23.2023. doi: 10.1523/ENEURO.0237-23.2023 (PMC10726287; doi:10.1523/ENEURO.0237-23.2023)

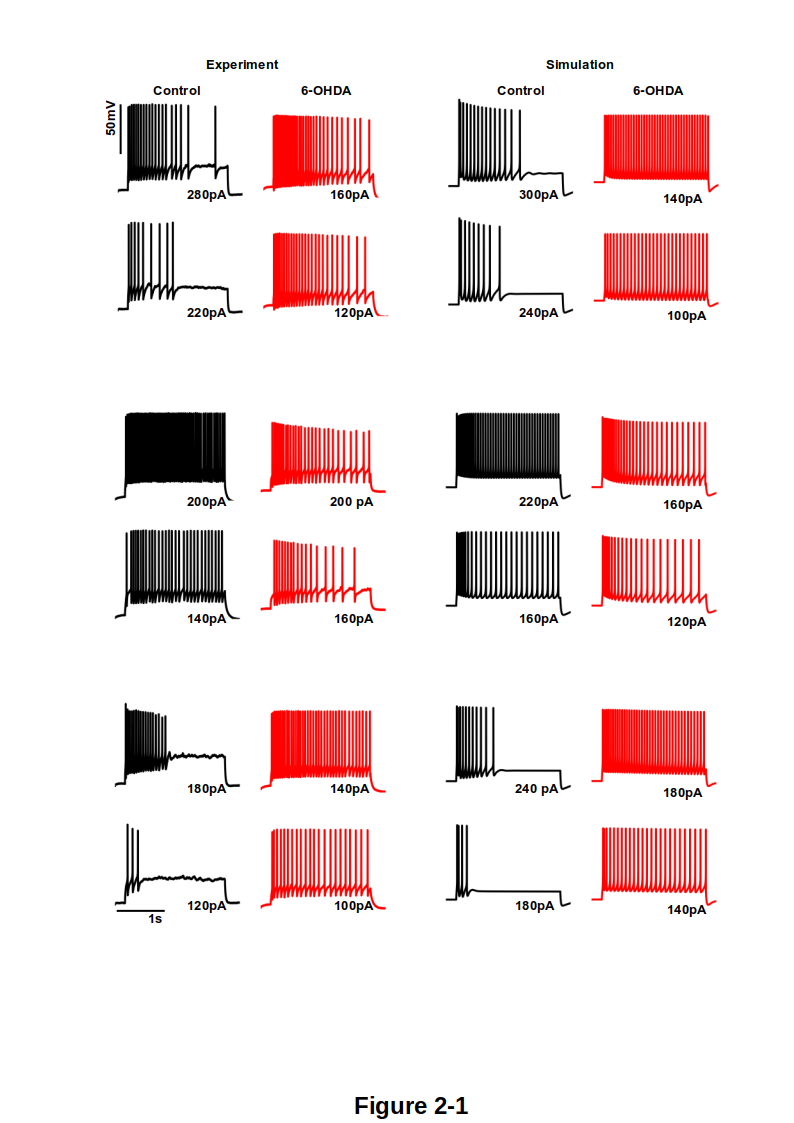

Supplement: Extended Data Figure 2-1 — Diversity of experimental and simulated responses of thalamocortical neurons from ventromedial motor thalamus in normal and parkinsonian states. Neuron responses to increasing depolarizing current injections for different neurons (n = 3) and models (n = 3), under the same experimental conditions as in Figure 2A. Both experimental and simulated recordings display variability in different action potential (AP) properties, such as AP amplitude, AHP depth, AP accommodation. Download Figure 2-1, TIF file. [file enu-eN-NWR-0237-23-s01.tif]

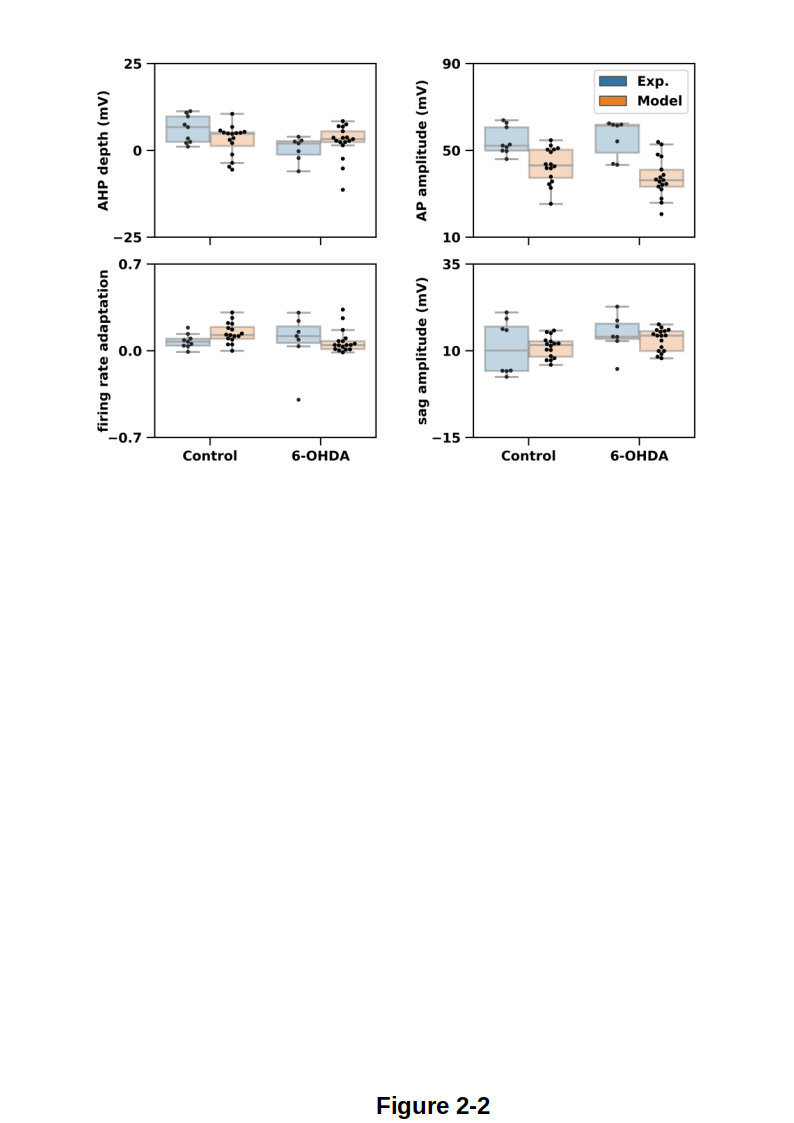

Supplement: Extended Data Figure 2-2 — Comparison of action potential properties between experimental and simulation traces. Experimental and simulation traces displayed similar firing properties. A subset of experimental and simulation traces is shown in Figure 1A and Extended Data Figure 1-1. Each data point (black) is associated with a single TC neuron (Control: n = 9 and 6-OHDA: n = 7 neurons) and TC neuron model (Control: n = 16 and 6-OHDA: n = 17 models). We compared after-hyperpolarization (AHP) depth (top, left), action potential (AP) amplitude (top, right), firing (rate) adaptation (bottom, left), and sag amplitudes (bottom, right) of TC neurons (Control: n = 9 and 6-OHDA: n = 7 neurons; blue) and TC neuron models (Control: n = 16 and 6-OHDA: n = 17 models; orange). The same firing properties were considered as a target during the model fitting (see also Materials and Methods) and are described in Table 2. We used protocol 1 (i.e., the same stimulation protocol as in Figure 1A; for details, see Materials and Methods) to measure AHP depth, AP amplitude, and firing adaptation, while we used protocol 3 for sag amplitudes, in both experiments and simulations. The values of AHP depth and AP amplitude were estimated using threshold amplitudes of step current, while we used the minimal current evoking at least five APs for firing rate adaptation. As the threshold current depends on the input resistance of neurons, it was estimated for each real neuron and model. To measure the sag amplitudes, we used a hyperpolarizing step current (−200 pA). Comparing experimental and simulation traces, we found that most firing features were statistically indistinguishable in both normal and parkinsonian conditions (Mann–Whitney, p > 0.04), except the AP amplitudes (Mann–Whitney, Control: p < 0.01; 6-OHDA: p < 0.001), which were lower in simulations than experiments (Control: 21%; 6-OHDA: 33%). Download Figure 2-2, TIF file [file enu-eN-NWR-0237-23-s02.tif]

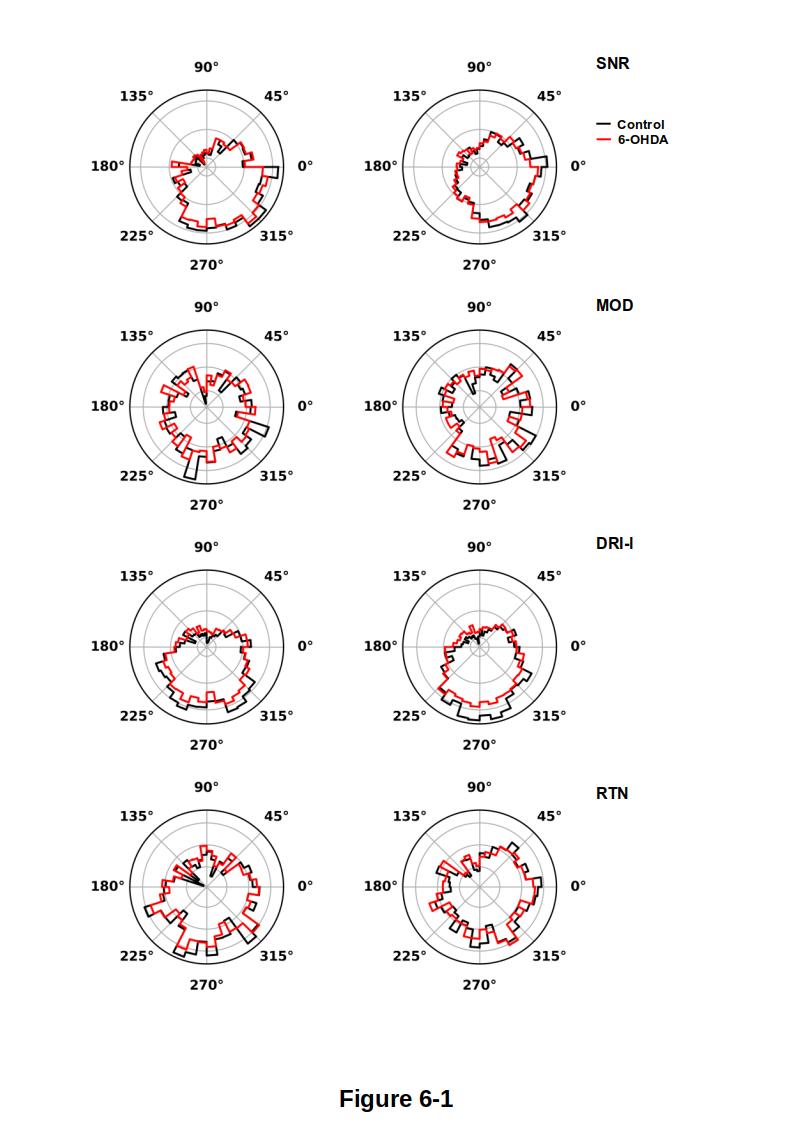

Supplement: Extended Data Figure 6-1 — β Modulation of different synaptic inputs individually. Related to Figure 6. Phase plots of the spiking activity for normal (black) and parkinsonian (red) models of thalamocortical (TC) neurons in presence of β modulation in the firing activity of substantia nigra reticulata (SNR), excitatory modulators (MOD), excitatory driver-like (DRI-l) inputs, and reticular thalamic nuclei (RTN). For each group of synaptic inputs, β modulation induces significant spike-phase locking in the activity of TC neuron models in both states (Rayleigh, p < 0.001). Each phase plot was obtained by averaging the responses of different TC neuron models (Control: n = 16; 6-OHDA: n = 17) and multiple simulations per model (n = 10). Compared to MOD and RTN inputs (128.1–154.9°), the circular SD of the spiking activity in TC neuron models achieved the lowest values with β modulation in SNR and DRI-l inputs (93.4–107.4°), suggesting that SNR and DRI-l inputs can induce the strongest spike-phase locking in TC neurons. Download Figure 6-1, TIF file. [file enu-eN-NWR-0237-23-s04.tif]

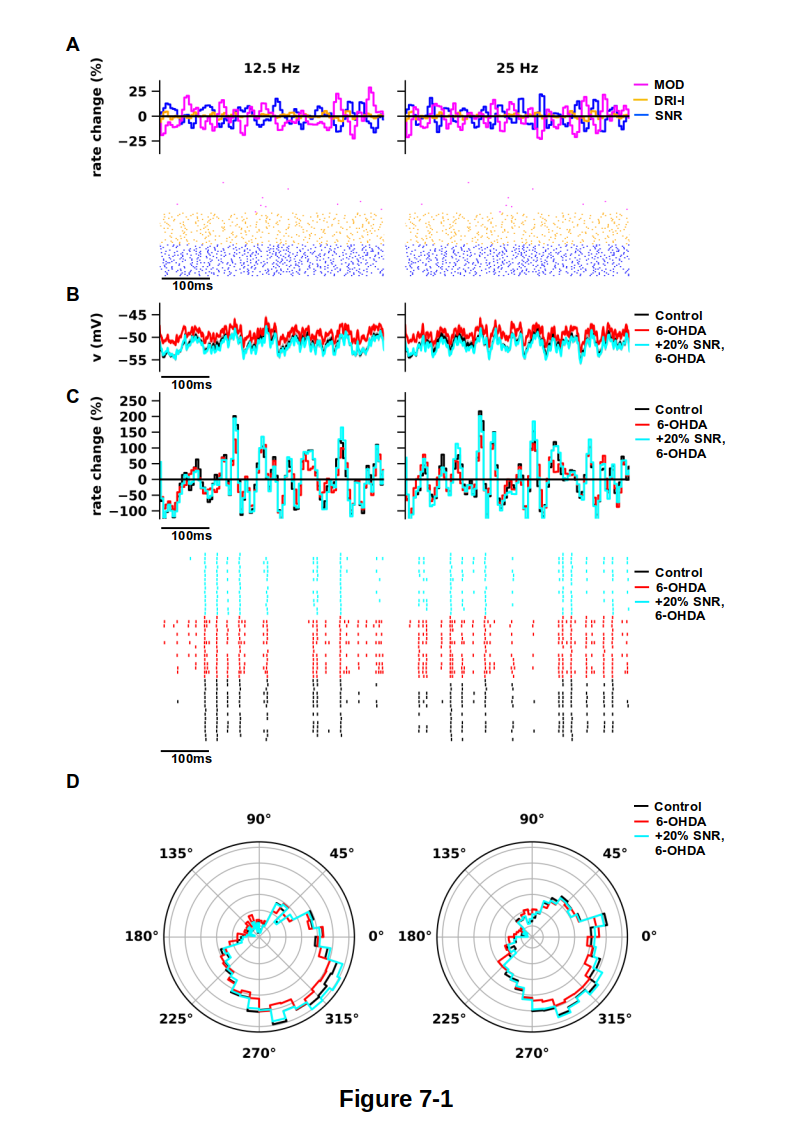

Supplement: Extended Data Figure 7-1 — β Modulation of inhibitory inputs from substantia nigra reticulata and excitatory driver-like inputs. β Modulation in the activity of substantia nigra reticulata (SNR) and driver-like inputs (DRI-l) at 12.5 Hz (left) and 25 Hz (right). Modulators (MOD) were not modulated. The oscillations in DRI-l were shifted by 180° with respect to SNR inputs. The effects of β modulation in SNR only are shown in Figure 7. A, Spike histograms show the relative variation of instantaneous firing rate to average versus time (top) and exemplificative raster plots (bottom) of the presynaptic activity for MOD (magenta), DRI-l (orange), and SNR (blue). The spike histograms were calculated from multiple simulations (n = 10). The raster plots of each input were a subset of total (n = 25). B, Somatic voltage traces of thalamocortical (TC) neuron models in normal (Control; black) and parkinsonian (6-OHDA) states, with regular (red) and increased (+20%; cyan) SNR conductance for parkinsonian models. Each curve was obtained by averaging the voltage traces generated by models (Control: n = 16; 6-OHDA: n = 17) and multiple simulations per model (n = 10). C, Spike histograms showing the relative variation of instantaneous firing rate to average versus time (top) and exemplificative rastergrams (bottom) of TC neuron activity in normal (Control; black) and parkinsonian (6-OHDA) states, with regular (red) and increased (+20%; cyan) SNR conductance for parkinsonian models. The spike histograms were calculated from the pooled responses of multiple models (Control: n = 16; 6-OHDA: n = 17) and multiple simulations per model (n = 10). Each trial was associated with a random seed that determines the subcellular distributions and activation timing (i.e., artificial spike trains) of synapses (for details, see Materials and Methods). The rastergrams show the activity of TC neuron models (n = 15) in normal (black) and parkinsonian states (red, cyan), obtained with a single simulation. D, Phase plots of the spi [file enu-eN-NWR-0237-23-s05.tif]

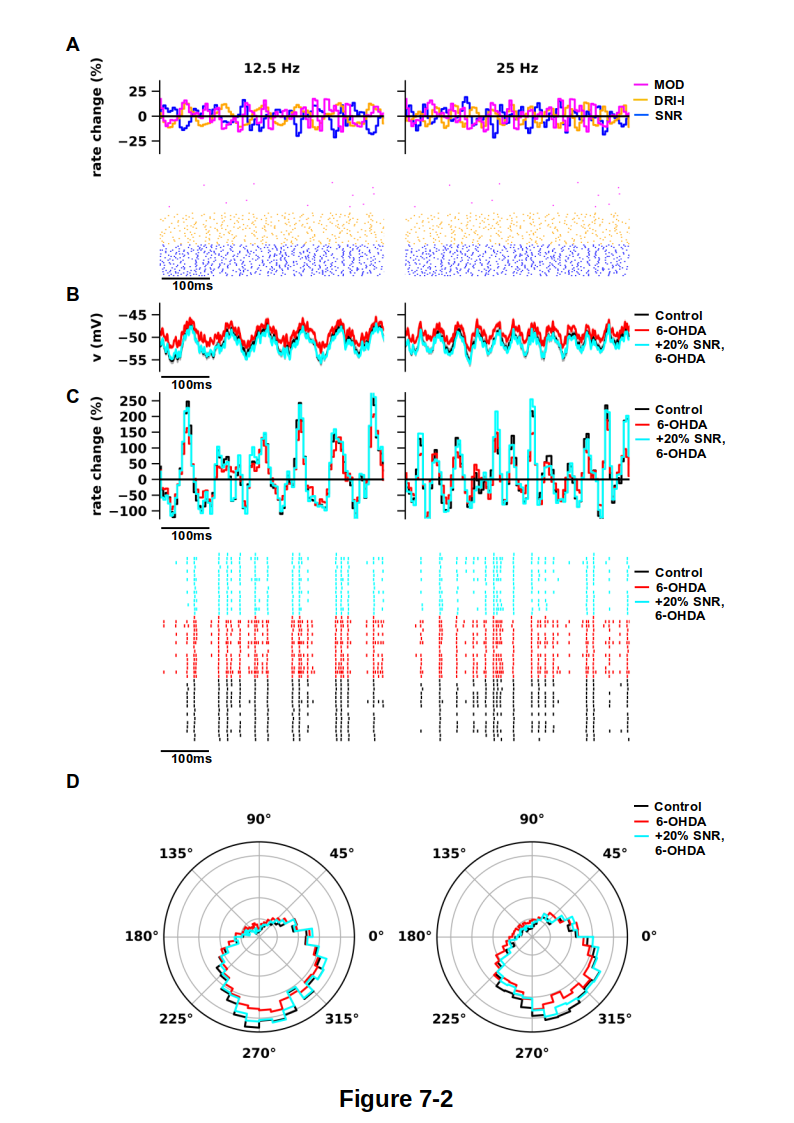

Supplement: Extended Data Figure 7-2 — β Modulation of inhibitory inputs from substantia nigra reticulata and excitatory modulators. β Modulation in the activity of substantia nigra reticulata (SNR) and excitatory modulators (MOD) at 12.5 Hz (left) and 25 Hz (right). Driver-like inputs (DRI-l) were not modulated. The oscillations in MOD were shifted by 180° with respect to SNR inputs. The effects of β modulation in SNR only are shown in Figure 7. A, Spike histograms show the relative variation of instantaneous firing rate to average versus time (top) and exemplificative raster plots (bottom) of the presynaptic activity for MOD (magenta), DRI-l (orange), and SNR (blue). The spike histograms were calculated from multiple simulations (n = 10). The raster plots of each input were a subset of total (n = 25). B, Somatic voltage traces of thalamocortical (TC) neuron models in normal (Control; black) and parkinsonian (6-OHDA) states, with regular (red) and increased (+20%; cyan) SNR conductance for parkinsonian models. Each curve was obtained by averaging the voltage traces generated by models (Control: n = 16; 6-OHDA: n = 17) and multiple simulations per model (n = 10). C, Spike histograms showing the relative variation of instantaneous firing rate to average versus time (top) and exemplificative rastergrams (bottom) of TC neuron activity in normal (Control; black) and parkinsonian (6-OHDA) states, with regular (red) and increased (+20%; cyan) SNR conductance for parkinsonian models. The spike histograms were calculated from the pooled responses of multiple models (Control: n = 16; 6-OHDA: n = 17) and multiple simulations per model (n = 10). Each trial was associated with a random seed that determines the subcellular distributions and activation timing (i.e., artificial spike trains) of synapses (for details, see Materials and Methods). The rastergrams show the activity of TC neuron models (n = 15) in normal (black) and parkinsonian states (red, cyan), obtained with a single simulation. D, Phase plots of the sp [file enu-eN-NWR-0237-23-s06.tif]

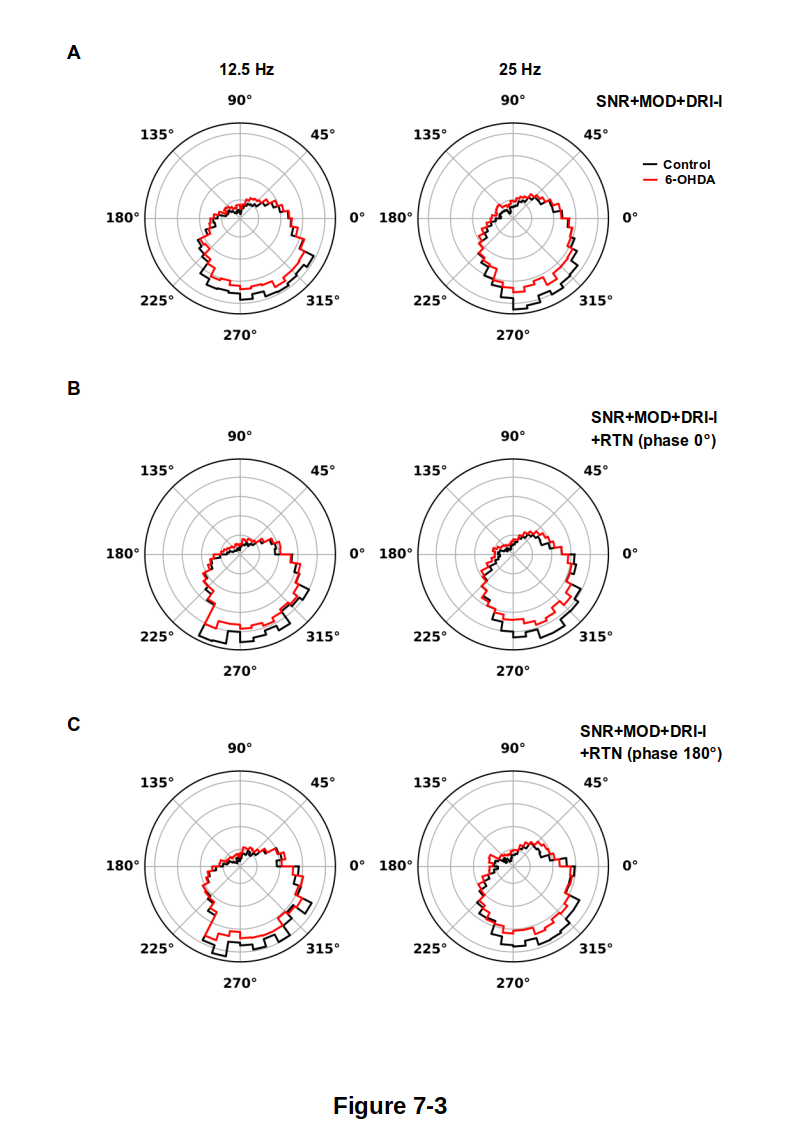

Supplement: Extended Data Figure 7-3 — β Modulation of inhibitory inputs from substantia nigra reticulata, excitatory modulators and driver-like inputs, and reticular thalamic nuclei. A, Phase plots of the spiking activity shown in Figure 7 for normal (black) and parkinsonian (red) models of thalamocortical neurons (Rayleigh, p < 0.001). The spike histograms were calculated from the pooled responses of different models (Control: n = 16; 6-OHDA: n = 17) and multiple simulations per model (n = 10). B, Same as in A with β modulation of reticular inputs in phase with inputs from substantia nigra reticulata (SNR; Rayleigh, p < 0.001). C, Same as in A with β modulation of reticular inputs shifted by 180° with respect to SNR inputs (Rayleigh, p < 0.001). Download Figure 7-3, TIF file. [file enu-eN-NWR-0237-23-s07.tif]

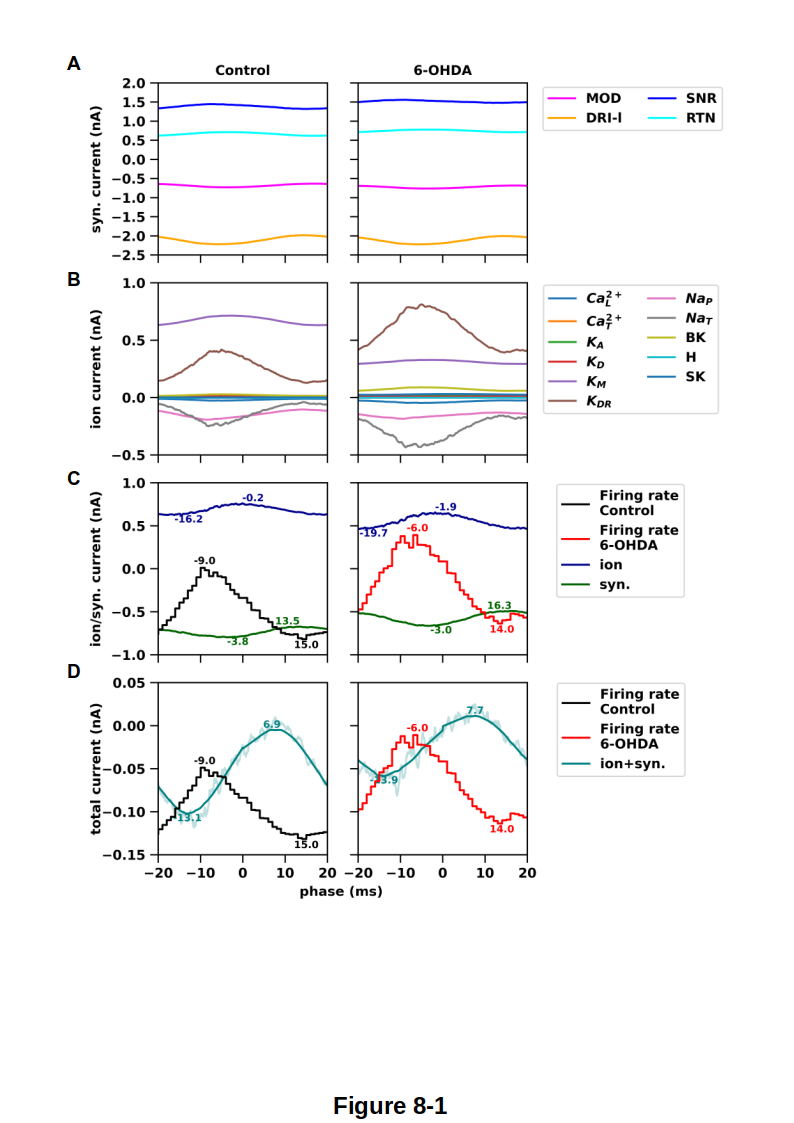

Supplement: Extended Data Figure 8-1 — Synaptic and membrane currents resulting from β modulation of synaptic inputs at 25 Hz. Same simulations as shown in Figure 8 with β modulation at 25 Hz, using the same configuration of inputs shown also in Figure 7, right. Download Figure 8-1, TIF file. [file enu-eN-NWR-0237-23-s08.tif]
